# Peroxide-cleavable linkers for antibody–drug conjugates†

**DOI:** 10.1039/d2cc06677g

**Published:** 2023-01-18

**Authors:** Nicola Ashman, Jonathan D. Bargh, Stephen J. Walsh, Ryan D. Greenwood, Arnaud Tiberghien, Jason S. Carroll, David R. Spring

**Affiliations:** a Yusuf Hamied Department of Chemistry, University of Cambridge Lensfield Road Cambridge CB2 1EW UK spring@ch.cam.ac.uk; b Cancer Research UK Cambridge Institute Robinson Way Cambridge CB2 ORE UK; c Oncology R&D, AstraZeneca Cambridge CB4 OWG UK; d Spirogen, Astrazeneca, The QMB Innovation Centre 42 New Rd London E1 2AX UK

## Abstract

Antibody–drug conjugates containing peroxide-cleavable arylboronic acid linkers are described, which target the high levels of reactive oxygen species (ROS) in cancer. The arylboronic acid linkers rapidly release a payload in the presence of hydrogen peroxide, but remain stable in plasma. Anti-HER2 and PD-L1 peroxide-cleavable ADCs exhibited potent cytotoxicity *in vitro*.

## Abstract 2

Peroxide-cleavable arylboronates are stable linkers for the generation of efficacious antibody–drug conjugates.

## Graphical abstract

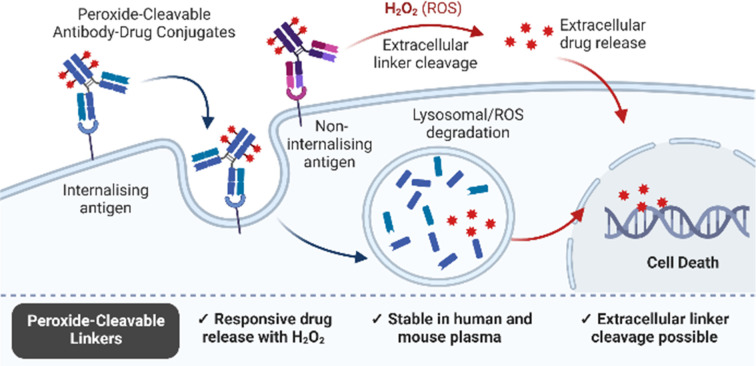

Antibody–drug conjugates (ADCs) are a class of oncology therapeutics that combine the high selectivity of monoclonal antibodies with the potency of small molecule cytotoxins. Their therapeutic success is reflected in the FDA approval of 14 ADCs, with over 100 others currently under clinical evaluation.^1^

The choice of antibody-payload linkage is essential in determining the selectivity and stability of ADC. For example, linkers containing reducible disulfides or acid-sensitive moieties have been shown to suffer from instability in circulation which leads to off-target toxicity.^2,3^ Enzyme-cleavable linkers are commonly employed, such as dipeptidic linkers targeting cathepsins that are upregulated in many cancer types.^4^ However, these dipeptide linkers are susceptible to hydrolysis by the Ces1C hydrolase present in mouse plasma, complicating pre-clinical ADC evaluation.^2,5^ Given the importance of linker technology for the success of ADCs, and the wide range of different cancer targets, there is need to explore new cleavable moieties with novel modes of action.

Cancer cells exhibit oxidative stress, and therefore often have elevated levels of reactive oxygen species (ROS) such as hydrogen peroxide (H_2_O_2_, the most long-lived ROS).^6^ The high ROS levels in cancer cells compared to normal cells has been exploited for the selective activation of ROS-sensitive small-molecule prodrugs,^7–11^ whereby boronic acid/ester moieties are either used to directly mask a key hydroxyl group of the parent drug, or are appended *via* a self-immolative benzyl spacer unit which disassembles following oxidation of the carbon–boron bond (Fig. 1).

**Fig. 1:**
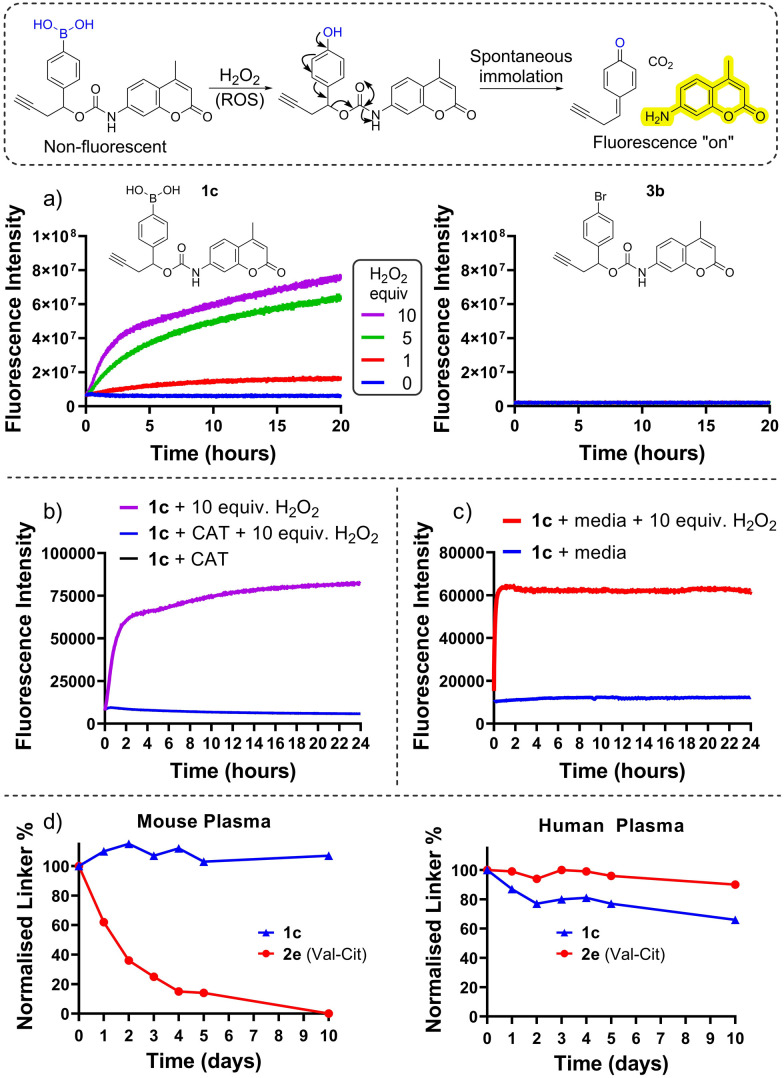
Illustration of the “turn-on” fluorescence from model linkers and general oxidation of boronic acids by H_2_O_2_ (top), (a) kinetics of AMC release from peroxide-cleavable 1c and non-cleavable 2c model linkers, (b) AMC release is prevented when co-incubated with catalase, (c) model linkers are stable in media (DMEM) in the absence of H_2_O_2_, (d) model linkers are stable in human and mouse plasma by HPLC.

Herein we describe the application of arylboronic acids for the development of peroxide-cleavable linkers for ADCs. For preliminary studies, model arylboronic linkers were synthesised comprising a fluorescent small molecule 7-amino 4-methyl coumarin (AMC) for convenient assessment of payload release kinetics and stability by fluorimetry (Fig. 1). Whilst non-fluorescent when connected by a carbamate bond, upon peroxide induced linker cleavage, free AMC is released which displays significantly enhanced fluorescence. Synthesis of arylboronic acid linker 1c was achieved by Miyaura borylation of 4-bromobenzaldehyde yielding 1a, followed by a zinc Barbier reaction with propargyl bromide to give benzylic alcohol 1b, containing an alkyne handle for later antibody functionalisation *via* copper-click chemistry (Fig. 2). AMC was then converted *in situ* to an isocyanate by triphosgene, for subsequent reaction with 1b and hydrolysis of the boronic ester to acid to afford 1c. A non-cleavable model linker 3b (Fig. 1(a) and Scheme S3, ESI†) was synthesised as a negative control, lacking the key boronic acid motif rendering it unable to release AMC by action of hydrogen peroxide. A valine-citrulline (Val-Cit) model linker 2e (Scheme S2, ESI†) was also synthesised to allow comparison of the stability of linkers to those commonly employed in ADCs.^12^

First, peroxide-cleavable model linker 1c was incubated with 10 equiv. H_2_O_2_ at 37 °C over 20 h (Fig. 1(a)). The increase in fluorescence over time confirmed that 1c displayed the desired reactivity with H_2_O_2_, and the lack of fluorescence increase without H_2_O_2_ confirmed that this was not due to generic instability. Non-cleavable linker 3c (Fig. 1(a)) displayed no temporal increase in fluorescence intensity when incubated with peroxide, highlighting that the boronic acid motif is key for the mechanism of AMC release. When co-incubated with H_2_O_2_ and ROS-scavenger catalase (CAT) no fluorescence increase was observed from 1c (Fig. 1(b)), highlighting that the release is mediated by H_2_O_2_.

To assess plasma stability, model linker 1c and analogous Val-Cit linker 2e were incubated with human and mouse plasma at 37 °C for 10 days (Fig. 1(d), and Tables SI-2–SI-5, ESI†) and monitored by HPLC. The boronic acid model linker was highly stable in both human and mouse plasma. In contrast, the Val-Cit dipeptide rapidly hydrolysed in mouse plasma presumably due to its susceptibility to the mouse Ces1c hydrolase.^2^

Finally, in the presence of unconditioned cell growth media (in the absence of cancer cells), 1c displayed no significant increase in fluorescence intensity over 4 days, in contrast to when co-incubated with 10 equiv. H_2_O_2_ (Fig. 1(c) and Fig. S16, ESI†). This highlights that the linkers are not appreciably unstable in cell media but are susceptible to cleavage in the presence of peroxide.

**Fig. 2:**
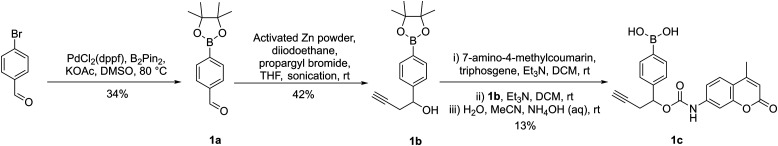
Synthetic route towards peroxide-cleavable model linker 1c.

Having confirmed model linker 1c was plasma stable and gave responsive cleavage with peroxide it was elaborated into an anti-HER2 ADC with trastuzumab. First, benzylic alcohol 1b was activated as a *para*-nitro phenyl carbonate for reaction with potent cytotoxin monomethyl auristatin E (MMAE), affording linker-payload 1e (Fig. 3(a)). Then, copper-catalysed azide–alkyne cycloaddition (CuAAC) of alkyne 1e and DVP-azide 4d yielded desired linker-payload 1f.

**Fig. 3:**
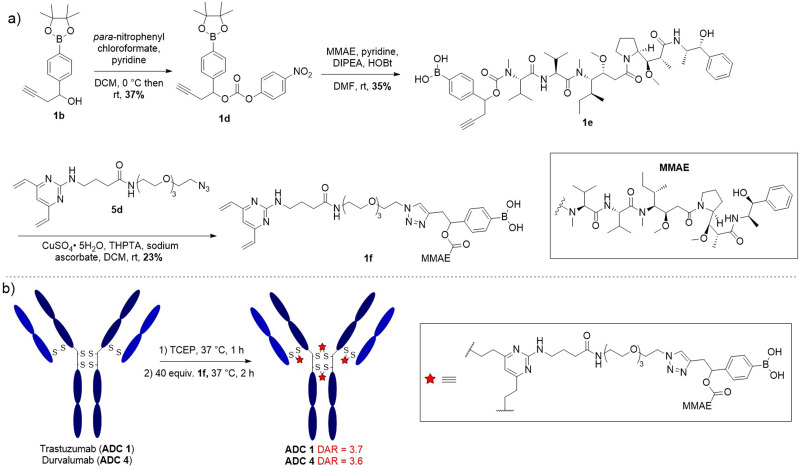
(a) Synthetic route towards DVP-linker-MMAE 1f; (b) bioconjugation conditions to final ADC 1 and ADC 4.

The DVP bioconjugation handle has been shown to generate highly stable ADCs with a drug-to-antibody (DAR) of 4 by rebridging the native interchain disulfide bonds.^13,14^ Accordingly, the disulfide bonds of trastuzumab were reduced with TCEP for 1 h, followed by reaction with DVP-linker-payload 1f to afford ADC 1 (Fig. 3(b)). Efficient conversion to the ADCs was confirmed by LC-MS, SDS-PAGE and hydrophobic interaction chromatography (HIC) (Fig. S5–S9, S1, S4, ESI†) and size-exclusion chromatography (SEC) analysis revealed minimal aggregation (<1%) (Fig. S2, ESI†). Val-Cit and non-cleavable ADCs (ADC 2 and ADC 3 respectively) were also synthesised for comparison of *in vitro* activity (Fig. S1, ESI†).

Having successfully generated ADCs 1–3, their *in vitro* cytotoxicity against HER2-positive (SKBR3 and BT474) and HER2-negative (MCF7) breast cancer cells were evaluated. Both antigen-positive and antigen-negative cancer cell lines are known to produce ROS which can freely diffuse into the extracellular media, due to increased oxidative stress. Accordingly, we observed that the peroxide-cleavable ADC 1 displayed potent dose-dependent cell killing in both HER2-positive and HER2-negative cell lines (Fig. 4(a)). This apparent lack of selectivity for antigen positive cells is due to prolonged incubation in media that contains ROS produced by the cancer cells. This is supported by the observation that non-cleavable ADC 3 and Val-Cit analogue ADC 4 which both lack the key peroxide-responsive motif, are non-toxic in HER2-negative cell lines.

**Fig. 4:**
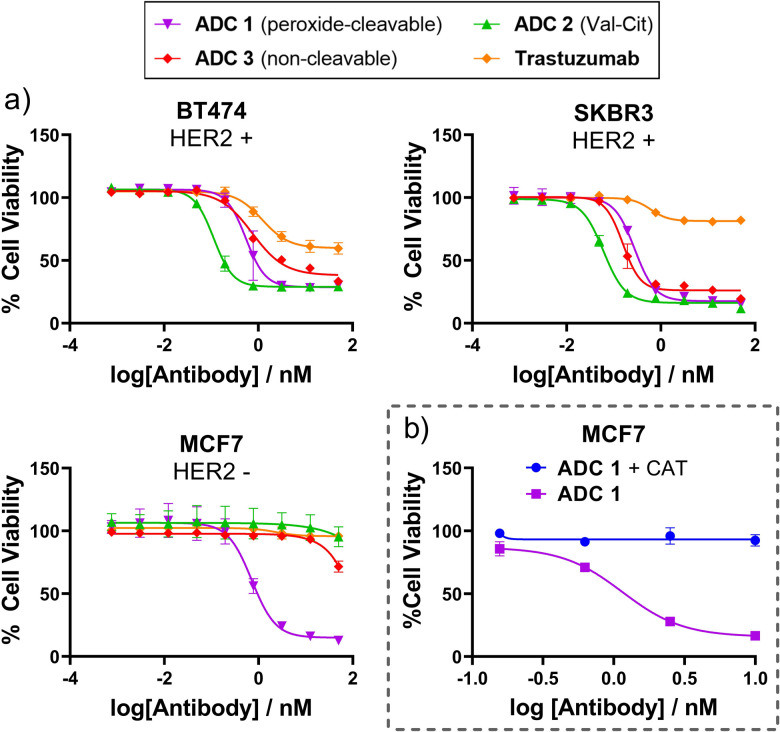
(a) *In vitro* cytotoxicity of ADC 1 in HER2+ and HER2− breast cancer cell lines; (b) ADC 1 becomes non-toxic to MCF7 cells when H_2_O_2_ is scavenged by catalase (CAT).

To verify the hypothesis that the toxicity of ADC 1 in HER2-MCF7 cells is a result of extracellular H_2_O_2_, we first confirmed the presence of extracellular H_2_O_2_ by Amplex™ red detection: MCF7 cells exhibited significantly higher fluorescence than the negative “no cell” control, indicating extracellular H_2_O_2_ (Fig. SI7, ESI†). Furthermore, when ADC 1 was co-incubated with H_2_O_2_ scavenger Catalase (CAT), MCF7 cell viability remained around 100% with 10 nM ADC treatment, compared to approx. 17% cell viability without CAT (Fig. 4(b)). Hence, H_2_O_2_ is crucial for the activity of peroxide-cleavable ADC 1 in HER2-MCF7 cells.

Given the apparent ability of the ROS-responsive ADC to kill cancer cells without requiring receptor-mediated internalisation, we envisaged the arylboronate linkers could be utilised for the generation of non-internalising ADCs. By incorporating an antibody selective for a non-internalising antigen target, after antibody–antigen binding, the high levels of ROS in the cancer extracellular matrix should enable extracellular payload release. This non-internalising mechanism of action may avoid some of the limitations associated with internalising ADCs such as inefficient internalisation, lack of suitable internalising antigen targets, and acquired resistance related to the internalisation process.^15,16^

Programmed cell death ligand-1 (PD-L1) overexpression has been identified in many malignancies and several antibodies which block the PD-L1-PD1 binding interaction have gained FDA approval. Recent work has also investigated the generation of PD-L1 ADCs.^17^

Given that some antibodies targeting PD-L1 do not internalise well/quickly,^16^ we sought to conjugate peroxide-cleavable linkers with anti-PD-L1 antibody durvalumab to generate a non-internalising ADC. Durvalumab was subjected to the bioconjugation conditions previously employed for trastuzumab (Fig. 3(b)), since they share the IgG_1_ structure. This afforded durvalumab ADCs 4–6 with average DARs close to 4 (determined by HIC, Fig. S4, ESI†). SEC showed minimal aggregation (Fig. S2, ESI†) and LC-MS confirmed formation of the desired antibody species (Fig. S10–S12, ESI†). The ADCs were then evaluated for their *in vitro* cytotoxicity in PD-L1-positive TNBC cell line MDA-MB-231, which was shown to possess extracellular ROS by Amplex™ red detection (Fig. S17, ESI†).

Gratifyingly, only peroxide-cleavable ADC 5 showed dose-dependent cell killing of PD-L1-positive MDA-MB-231 cells *in vitro*, whereas the non-cleavable and Val-Cit analogues (ADC 6 and 7), lacking the ROS-responsive linker, showed no activity up to 50 nM (Fig. 5). This indicates that the ADCs were not internalised and catabolised intracellularly, but extracellular cleavage of the arylboronate linker of ADC 4 had occurred to allow extracellular payload release. In PD-L1-negative MCF7 cells,^18^ADC 4 also displayed potent cell killing (Fig. 5), highlighting that internalisation was not required for payload release but linker cleavage was enabled by prolonged incubation in cell media containing H_2_O_2_. Furthermore, when treated with H_2_O_2_ scavenger CAT, ADC 4 was non-toxic to both PD-L1-negative MCF7 cells and PD-L1-positive MDA-MB-231 cells (Fig. 5(b)). This suggests limited internalisation of ADC 4 into PD-L1 positive MDA-MB-231 cells, since it is expected that following internalisation and antibody degradation, the Cys-Linker-MMAE catabolite would retain cytotoxicity.^19^

**Fig. 5:**
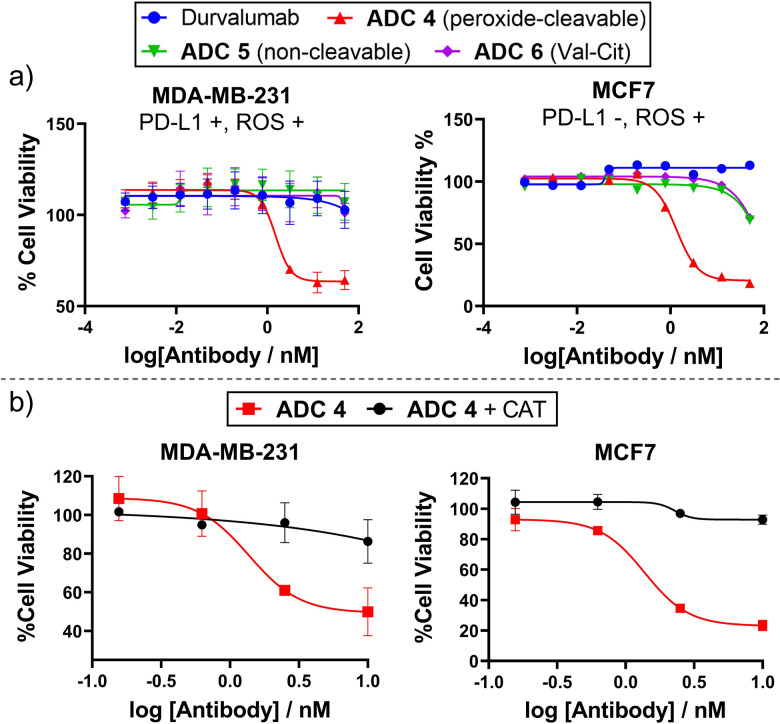
(a) *In vitro* cytotoxicity of Durvalumab ADCs 4–6 in PD-L1 + and PD-L1 – breast cancer cell lines; (b) ADC 4 becomes non-toxic to MCF7 and MDA-MB-231 cells when H_2_O_2_ is scavenged by catalase (CAT).

In summary, we have demonstrated that peroxide-cleavable arylboronic acids are plasma stable ADC linkers which efficiently react with hydrogen peroxide. Peroxide-cleavable anti-HER2 and anti-PD-L1 ADCs were successfully generated and displayed potent cytotoxicity *in vitro*. An extracellular mechanism of action has been postulated from the potent cell killing of antigen-negative cells by ROS-responsive ADC 1, but not with non-cleavable/Val-Cit analogues or when H_2_O_2_ was scavenged by catalase.

It is anticipated that peroxide-cleavable linkers could be a versatile linker choice for the development of ADCs with internalising or non-internalising mechanisms of action.

N. Ashman – investigation, formal analysis, writing original draft. J. D. Bargh – conceptualization, supervision, writing – review and editing. S. J. Walsh, R. D. Greenwood, A. Tiberghien & D. R. Spring – supervision, writing – review and editing.

N. Ashman acknowledges a studentship from AstraZeneca. The Spring lab acknowledges support from the EPSRC, BBSRC, MRC and Royal Society. N. Ashman is grateful to the GB lab for allowing access to the Pherastar plate reader and to Prof. Michael P. Murphy for advice on measuring ROS. Table of contents entry created with Biorender.com.

## Conflicts of interest

J. D. B. is now an employee of AstraZeneca. S. J. W. and A. T. are now employed by Bicycle Therapeutics.

## Supplementary Material

CC-059-D2CC06677G-s001
