# Supplementary material for: Peroxide-cleavable linkers for antibody–drug conjugates
Source: Chem Commun (Camb). 2023 Jan 18;59(13):1841–4. doi: 10.1039/d2cc06677g (PMC9910056; doi:10.1039/d2cc06677g)
Supplement: CC-059-D2CC06677G-s001 [file CC-059-D2CC06677G-s001.pdf]

Electronic Supplementary Material (ESI) for ChemComm.
This journal is © The Royal Society of Chemistry 2023

Supporting Information: Peroxide-Cleavable Linkers for Antibody-Drug Conjugates

Contents

General Experimental 2
Chemical Synthesis 3
Bioconjugation 12
SDS-PAGE 13
Size-Exclusion Chromatography 16
Hydrophobic Interaction Chromatography 17
Protein LC-MS 19
Peroxide-Cleavage Studies 24
Stability Studies 25
Cell Lines 29
In Vitro Cytotoxicity 30
NMR Spectra 32

General Experimental

All solvents and reagents were used as received unless otherwise stated. Ethyl acetate, methanol, dichloromethane, acetonitrile and toluene were distilled from calcium hydride. Petroleum ether (PE) refers to the fraction between 40–60 °C upon distillation. Tetrahydrofuran (THF) was dried using Na wire and distilled from a mixture of lithium aluminium hydride and calcium hydride with triphenylmethane as indicator. Non-aqueous reactions were conducted under a stream of dry nitrogen using oven-dried glassware. Temperatures of 0 °C were maintained using an ice-water bath. Room temperature (rt) refers to ambient temperature. Yields refer to spectroscopically and chromatographically pure compounds unless otherwise stated. Reactions were monitored by thin layer chromatography (TLC) or liquid chromatography mass spectroscopy (LC-MS). TLC was performed using glass plates pre-coated with Merck silica gel 60 F254 and visualised by quenching of UV fluorescence (λ_{max} = 254 nm) or by staining with potassium permanganate. Retention factors (Rf) are quoted to 0.01. Flash column chromatography was carried out using Merck 9385 Kieselgel 60 SiO_2 (230-400 mesh) or Combiflash Rf200 automated chromatography system with Redisep® normal-phase silica flash columns (35−70 µm) or Redisep® reverse-phase C18-silica flash columns (20-40 µm). Analytical high performance liquid chromatography (HPLC) was performed on Agilent 1260 Infinity machine, using a Supelcosil™ ABZ+PLUS column (150 mm × 4.6 4 mm, 3 µm) with a linear gradient system (solvent A: 0.05% (v/v) TFA in H_2O; solvent B: 0.05% (v/v) TFA in MeCN) over 20 min at a flow rate of 1 mL/min, and UV detection (λ_{max} = 220 – 254 nm). Infrared (IR) spectra were recorded neat on a Perkin-Elmer Spectrum One spectrometer with internal referencing. Selected absorption maxima (ν_{max}) are reported in wavenumbers (cm^{-1}). Proton and carbon nuclear magnetic resonance (NMR) were recorded using an internal deuterium lock on Bruker DPX-400 (400 MHz, 101 MHz), Bruker Avance 400 QNP (400 MHz, 101 MHz), Bruker Avance 500 Cryo Ultrashield (500 MHz, 126 MHz) and 600 MHz Bruker Avance 600 BBI (600 MHz). Tetramethylsilane was used as an internal standard. In proton NMR, chemical shifts (δH) are reported in parts per million (ppm), to the nearest 0.01 ppm and are referenced to the residual non-deuterated solvent peak ($CDCl_3$: 7.26, DMSO-d_6: 2.50, CD_3OD: 3.31, acetone-d_6: 2.05). Coupling constants (J) are reported in Hertz (Hz) to the nearest 0.1 Hz. Data are reported as follows: chemical shift, multiplicity (s = singlet; d = doublet; t = triplet; q = quartet; qn = quintet; sep = septet; m = multiplet; br = broad or as a combination of these, e.g. dd, dt etc.), integration and coupling constant(s). In carbon NMR, chemical shifts (δC) are quoted in ppm, to the nearest 0.1 ppm, and are referenced to the residual non-deuterated solvent peak ($CDCl_3$: 77.16, DMSO-d_6: 39.52, acetone-d_6: 19.84, 206.26). High resolution mass spectrometry (HRMS) measurements were recorded with a Micromass Q-TOF mass spectrometer or a Waters LCT Premier Time of Flight mass spectrometer. Mass values are reported within the error limits of ±5 ppm mass units. ESI refers to the electrospray ionisation technique. Protein LC–MS was performed on a Xevo G2-S TOF mass spectrometer coupled to an Acquity UPLC system using an Acquity UPLC BEH300 C4 column (1.7 µm, 2.1 × 50 5 mm). H_2O with 0.1% formic acid (solvent A) and 95% MeCN and 5% water with 0.1% formic acid (solvent B), were used as the mobile phase at a flow rate of 0.2 mL/min. The gradient was programmed as follows: 95% A for 0.93 min, then a gradient to 100% B over 4.28 min, then 100% B for 1.04 minutes, then a gradient to 95% A over 1.04 min. The electrospray source was operated with a capillary voltage of 2.0 kV and a cone voltage of 40 V, 120 V or 150 V. Nitrogen was used as the desolvation gas at a total flow of 850 L/h. Total mass spectra were reconstructed from the ion series using the MaxEnt algorithm preinstalled on MassLynx software (v4.1 from Waters) according to the manufacturer's instructions. Trastuzumab and durvalumab samples were deglycosylated with PNGase F (New England Biolabs) prior to LC-MS analysis. Fluorescence was measured with a Pherastar FS plate reader using a 350/460 optic module or a CLARIOstar microplate reader.

Chemical Synthesis

Unsubstituted Model Linker **1c**

Scheme S1: Synthesis of unsubstituted AMC model linker 1c

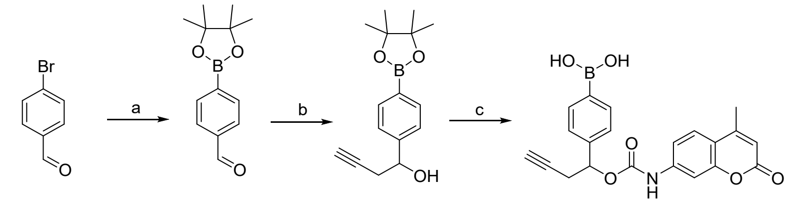

1a **1b** **1c**

Reagents and conditions: a) $PdCl_2$(dppf), B_2Pin_2, KOAc, DMSO, 80 °C, 3.5 h, 34%; b) Activated Zn powder, diiodoethane, propargyl bromide, THF, sonication, rt, overnight, 42%; c) AMC, triphosgene, Et_3N, CH_2Cl_2, rt, overnight, then H_2O:MeCN:NH_4OH (aq), rt, several days, 13%.

4-(4,4,5,5-tetramethyl-1,3,2-dioxaborolan-2-yl)benzaldehyde (1a)

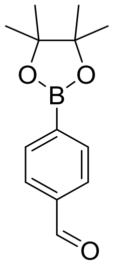

$PdCl_2$(dppf) (134.0 mg, 0.16 mmol), B_2Pin_2 (1.51 g, 5.95 mmol) and KOAc (1.59 g, 16.2 mmol) were suspended in DMSO (20 mL) and 4-bromobenzaldehyde (994 mg, 5.37 mmol) was added. The mixture was heated at 80 °C for 3.5 h, cooled to rt, then filtered through a pad of celite. The filtrate was concentrated *in vacuo* then purified by flash column chromatography (FCC) (5% EtOAc in PE) yielding 4-(4,4,5,5-tetramethyl-1,3,2-dioxaborolan-2-yl)benzaldehyde, **1a** (427 mg, 1.84 mmol, 34%) as a yellow oil.

R_f = 0.36 (2:1 EtOAc:PE); **^{1}H NMR** (400 MHz, $CDCl_3$): δ 9.95 (s, 1H), 7.88 (d, 2H, *J* = 7.7 Hz), 7.77 (d, 2H, *J* = 7.7 Hz), 1.28 (s, 12H); **^{13}C NMR** (101 MHz, $CDCl_3$): δ 192.4, 138.1, 135.2, 128.6, 84.2, 24.8.

These data are consistent with those previously reported.[1]

1-(4-(4,4,5,5-tetramethyl-1,3,2-dioxaborolan-2-yl)phenyl)but-3-yn-1-ol (1b)

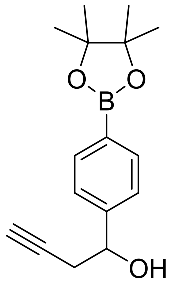

4-(4,4,5,5-tetramethyl-1,3,2-dioxaborolan-2-yl)benzaldehyde (**1a**) (199 mg, 0.857 mmol), activated Zn powder (282 mg, 4.31 mmol), diiodoethane (278 mg, 0.879 mmol) and propargyl bromide (80 wt% in toluene) (140 µL, 1.26 mmol) were suspended in THF (5 mL) and sonicated at rt overnight. 1N HCl (30 mL) was then added to the reaction mixture and was extracted with EtOAc (4 x 30 mL). The combined organic phases were washed with brine (30 mL), dried over Na_2SO_4 and concentrated *in vacuo*. The

crude residue was purified by FCC (40% EtOAc in PE) yielding 1-(4-(4,4,5,5-tetramethyl-1,3,2-dioxaborolan-2-yl)phenyl)but-3-yn-1-ol, **1b** (98.1 mg, 0.360 mmol, 42%) as a yellow oil.

R_f = 0.64 (50% EtOAc in PE); **^{1}H NMR** (400 MHz, $CDCl_3$): δ 7.80 (d, 2H, *J* = 7.9 Hz), 7.39 (d, 2H, *J* = 8.0 Hz), 4.89 (t, 1H, J = 5.8 Hz), 2.64 (m, 2H), 2.06 (t, 1H, *J* = 2.6 Hz) 1.34 (s, 12H); **^{13}C NMR** (101 MHz, $CDCl_3$): δ 145.5, 135.0, 125.0, 83.8, 80.5, 72.3, 71.0, 29.4, 24.9; **IR** ν_{max} /cm^{-1}: 3430br, 3288w, 2978w, 2931w, 1612m, 1514w; **HRMS (ESI)**: m/z calcd for [M+H]+ : 273.1657; found: 273.1661.

1-(4-(4,4,5,5-tetramethyl-1,3,2-dioxaborolan-2-yl)phenyl)but-3-yn-1-yl (4-methyl-2-oxo-2H-chromen-7-yl)carbamate (1c)

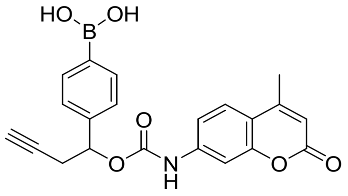

7-Amino-4-methylcoumarin (161 mg, 0.919 mmol) in CH_2Cl_2 (20 mL) was added triphosgene (114 mg, 0.383 mmol) then Et_3N (282 µL, 2.02 mmol). After 15 mins, 1-(4-(4,4,5,5-tetramethyl-1,3,2-dioxaborolan-2-yl)phenyl)but-3-yn-1-ol (**1b**) (250 mg, 0.919 mmol) was added. After 1 h, additional Et_3N (128 µL, 0.918 mmol) was added and the mixture left to stir at rt overnight. H_2O (1 mL) was then added to the reaction mixture and then concentrated *in vacuo*. The residue was suspended in a mixture of MeCN:H_2O:NH_4OH (aq) until basic by pH paper. After stirring for several days, the mixture was concentrated *in vacuo* and purified by reverse-phase FCC (10-80% solvent B in solvent A. Solvent A: H_2O. Solvent B: MeCN), yielding 1-(4-(4,4,5,5-tetramethyl-1,3,2-dioxaborolan-2-yl)phenyl)but-3-yn-1-yl (4-methyl-2-oxo-2H-chromen-7-yl)carbamate (**1c**) (45.0 mg, 0.115 mmol, 13%) as a white solid.

^{1}H NMR (400 MHz, acetone-d_6): δ 8.01 (s, 1H), 7.88 (d, 2H, *J* = 8.1 Hz), 7.69 (d, 1H, *J* = 8.7 Hz), 7.64 (d, 1H, *J* = 2.1 Hz), 7.51-7.52 (dd, 1H, *J* = 8.7, 2.1 Hz), 7.46 (d, 2H, *J* = 8.0 Hz), 6.16 (d, 1H, *J* = 1.1 Hz), 5.93 (t, 1H, *J* = 6.5 Hz), 2.82-2.89 (m, 2H), 2.44 (m, 4H); **^{13}C NMR** (101 MHz, acetone-d_6): δ 160.6, 155.1, 153.3, 153.1, 143.3, 142.0, 134.8, 126.4, 126.4, 114.8, 113.0, 105.6, 78.9, 75.1, 72.0, 26.6, 18.1. **IR** ν_{max}/cm^{-1}: 3293br, 1736m, 1682m, 1612m, 1366s; **HRMS (ESI)**: m/z calcd for [M+H]+ : 392.1305; found: 392.1312.

Val-Cit Model linker **2e**

Scheme S2: Synthesis of Val-Cit AMC model linker 2e

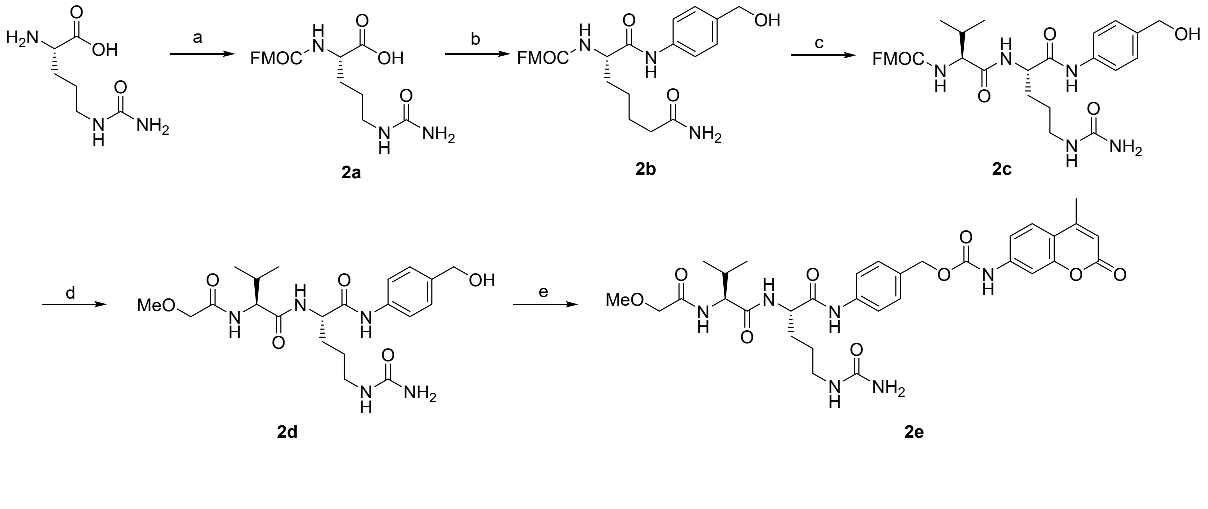

Reagents and conditions: a) FMOC-Cl, $NaHCO_3$, DME/H_2O, rt, overnight, 64%; b) 4-aminobenzyl alcohol, HATU, DIPEA, DMF, rt, overnight, 68%; c) i) Et_3N, DMF, rt, overnight, ii) FMOC-Val-OSu, DMF, 1 h, 54%; d) i) Et_3N, DMF, rt, overnight, ii) 0 °C, methoxyacetyl chloride, Et_3N, then rt, 1 h, 41%, e) AMC, triphosgene, Et_3N, CH_2Cl_2, rt, 18 h, 61%.

(S)-2-((((9H-fluoren-9-yl)methoxy)carbonyl)amino)-5-ureidopentanoic acid (2a)

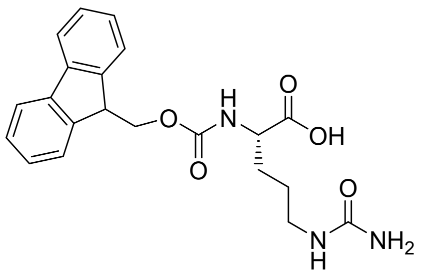

Citrulline (499 mg, 2.85 mmol) in H_2O (24 mL) was added $NaHCO_3$ (479 mg, 5.71 mmol). After 1 h at rt, FMOC-Cl (670 mg, 2.59 mmol) in DME (12 mL) was added. After stirring at rt overnight, DME was removed under vacuum and the mixture was extracted with EtOAc (3 x 30 mL). The aqueous layer was then acidified with 1 N HCl (15 mL). The aqueous phase was extracted with 10% iPrOH:EtOAc (3 x 50 mL) and then the combined organic phases dried over Na_2SO_4 then concentrated *in vacuo*. Et_2O (20 mL) was added to the clear viscous liquid, and the resulting white precipitate was collected by vacuum filtration, with successive washings with Et_2O, yielding (S)-2-((((9H-fluoren-9-yl)methoxy)carbonyl)amino)-5-ureidopentanoic acid **(2a)** (724 mg, 1.82 mmol, 64%) as a white solid.

^{1}H NMR (400 MHz, DMSO-d$_6$): δ 12.58 (br s), 7.90 (d, 2H, *J* = 7.5 Hz), 7.73 (d, 2H, *J* = 7.4 Hz), 7.67 (d, 1H, *J* = 8.0 Hz), 7.43 (app t, 2H, *J* = 7.4 Hz), 7.34 (app t, 2H, *J* = 7.3 Hz), 5.94 (br t, 1H), 5.38 (br s, 2H), 4.23-4.29 (m, 3H), 3.93-3.94 (m, 1H), 2.94-2.98 (m, 2H), 1.69-1.72 (m, 1H), 1.53-1.62 (m, 1H), 1.42-1.45 (m, 2H); **^{13}C NMR** (101 MHz, DMSO-d$_6$): δ 173.9, 158.7, 156.1, 143.8, 140.7, 127.6, 127.1, 125.3, 120.1, 65.6, 53.7, 46.6, 28.2, 26.8.

These data correspond to those reported in the [1].[2]

(9H-fluoren-9-yl)methyl(S)-(1-((4-(hydroxymethyl)phenyl)amino)-1-oxo-5-ureidopentan-2-yl)carbamate (2b)

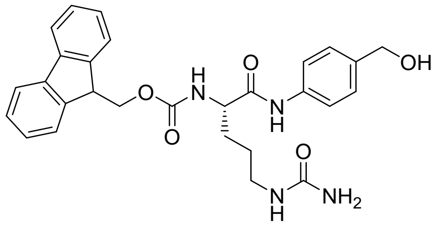

(S)-2-((((9H-fluoren-9-yl)methoxy)carbonyl)amino)-5-ureidopentanoic acid **(2a)** (720 mg, 1.81 mmol) and 4-aminobenzyl alcohol (672 mg, 5.45 mmol) in DMF (18 mL) was added DIPEA (317 µL, 1.82 mmol) and stirred at rt for 15 mins. Then, HATU (760 mg, 2.00 mmol) was added and the reaction mixture was stirred in the dark overnight. The reaction mixture was then concentrated *in vacuo* and purified by FCC (2-10% MeOH CH_2Cl_2), yielding (9H-fluoren-9-yl)methyl (S)-(1-((4-(hydroxymethyl)phenyl)amino)-1-oxo-5-ureidopentan-2-yl)carbamate/ FMOC-Cit-PAB-OH **(2b)** (623 mg, 1.24 mmol, 68%) as a pale orange solid.

^{1}H NMR (400 MHz, DMSO-d$_6$): δ 9.99 (s, 1H), 7.89 (d, 2H, *J* = 7.5 Hz), 7.75 (m, 2H), 7.67 (d, 1H, *J* = 7.9 Hz), 7.56 (d, 2H, *J* = 8.1 Hz), 7.42 (app t, 2H, *J* = 7.3 Hz), 7.34 (m, 2H), 7.24 (d, 2H, *J* = 8.1 Hz), 6.00 (br t,

1H), 5.43 (br s, 2H), 4.44 (s, 2H), 4.15-4.27 (m, 4H), 2.93-3.07 (m, 2H), 1.57-1.72 (m, 2H), 1.35-1.53 (m, 2H); **^{13}C NMR** (101 MHz, DMSO-d$_6$): δ 171.0, 158.9, 156.1, 143.9, 140.7, 137.6, 137.4, 127.7, 127.1, 126.9, 125.4, 120.1, 118.1, 65.7, 62.6, 55.0, 46.7, 38.6, 29.3, 26.9.

These data correspond to those reported in the literature.[2]

(9H-fluoren-9-yl)methyl ((S)-1-(((S)-1-((4-(hydroxymethyl)phenyl)amino)-1-oxo-5-ureidopentan-2-yl)amino)-3-methyl-1-oxobutan-2-yl)carbamate (2c)

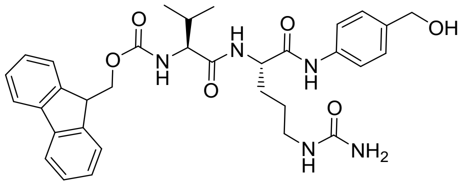

(9H-fluoren-9-yl)methyl (S)-(1-((4-(hydroxymethyl)phenyl)amino)-1-oxo-5-ureidopentan-2-yl)carbamate/ FMOC-Cit-PAB-OH **(2b)** (376 mg, 0.748 mmol) in DMF (4 mL) was added Et_3N (2 mL) and stirred at rt overnight. DMF and Et_3N were removed under a stream of N_2, then DMF (6 mL) was added followed by FMOC-Val-OSu (359 mg, 0.822 mmol) and the mixture stirred at rt for 1 h. The reaction mixture was concentrated by a stream of N_2 then purified by FCC (2-10% MeOH CH_2Cl_2) to yield (9H-fluoren-9-yl)methyl ((S)-1-(((S)-1-((4-(hydroxymethyl)phenyl)amino)-1-oxo-5-ureidopentan-2-yl)amino)-3-methyl-1-oxobutan-2-yl)carbamate/ FMOC-Val-Cit-PAB-OH **(2c)** (244 mg, 0.406 mmol, 54%) as a pale pink solid.

^{1}H NMR (400 MHz, DMSO-d$_6$): δ 10.00 (s, 1H), 8.11 (d, 1H, *J* = 7.5 Hz), 7.88 (d, 2H, *J* = 7.5 Hz), 7.76-7.72 (m, 2H), 7.55 (d, 2H, *J* = 8.1 Hz), 7.44-7.39 (m, 3H), 7.34-7.30 (m, 2H), 7.23 (d, 2H, *J* = 8.1 Hz), 6.05 (t, 1H, *J* = 5.2 Hz), 5.43 (s, 2H), 5.12 (br t, 1H), 4.43-4.42 (m, 3H), 4.30-4.22 (m, 3H), 3.95-3.91 (m, 1H), 3.04-2.89 (m, 2H), 2.02-1.98 (m, 1H), 1.75-1.67 (m, 1H), 1.63-1.58 (m, 1H), 1.49-1.33 (m, 2H), 0.89-0.85 (m, 6H); **^{13}C NMR** (101 MHz, DMSO-d$_6$): δ 171.3, 170.5, 159.0, 156.2, 144.0, 143.8, 140.8, 137.5, 127.7, 127.1, 127.0, 125.4, 120.1, 118.9, 65.8, 62.7, 60.2, 53.1, 46.7, 30.7, 29.6, 26.8, 19.3, 18.3.

These data correspond to those reported in the literature.[3]

(S)-N-(4-(hydroxymethyl)phenyl)-2-((S)-2-(2-methoxyacetamido)-3-methylbutanamido)-5-ureidopentanamide (2d)

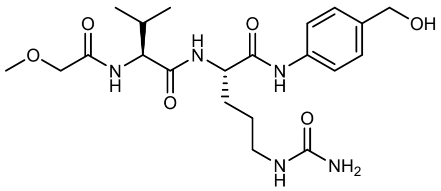

(9H-fluoren-9-yl)methyl ((S)-1-(((S)-1-((4-(hydroxymethyl)phenyl)amino)-1-oxo-5-ureidopentan-2-yl)amino)-3-methyl-1-oxobutan-2-yl)carbamate/ FMOC-Val-Cit-PAB-OH **(4c)** (275 mg, 0.458 mmol) in DMF (2 mL) was added Et_3N (1.30 mL, 9.32 mmol) and stirred at rt overnight. The reaction mixture was concentrated by a stream of N_2, then DMF (1 mL) was added and the solution cooled to 0 °C. Methoxyacetyl chloride (54.0 µL, 0.591 mmol) was then added, followed by Et_3N (83.0 µL, 0.595 mmol). After 30 mins, the reaction was warmed to rt, then stirred for 1 h. The reaction mixture was then quenched with H_2O (1 mL) and concentrated under a stream of N_2, then purified by FCC (10-15% MeOH CH_2Cl_2) yielding (S)-*N*-(4-(hydroxymethyl)phenyl)-2-((S)-2-(2-methoxyacetamido)-3-methylbutanamido)-5-ureidopentanamide **(2d)** (80.0 mg, 0.177 mmol, 41%) as a pale pink solid.

^{1}H NMR (400 MHz, DMSO-d$_6$) δ 9.98 (s, 1H), 8.31 (d, 1H, *J* = 7.3 Hz), 7.48-7.53 (m, 2H), 7.22 (d, 2H, *J* = 8.2 Hz), 6.05 (t, 1H, *J* = 5.6 Hz), 5.43 (br s, 2H), 5.18 (t, 1H, *J* = 5.5 Hz), 4.34-4.43 (m, 3H), 4.28 (m, 1H), 3.86 (s, 2H), 3.32 (s, 3H), 2.93-3.03 (m, 2H), 1.97-2.02 (m, 1H), 1.56-1.72 (m, 2H), 1.34-1.46 (m, 2H), 0.85 (d, 3H, *J* = 6.7 Hz), 0.81 (d, 3H, *J* = 6.7 Hz); **^{13}C NMR** (101 MHz, DMSO-d$_6$) δ 171.0, 170.5, 169.1, 159.2, 137.6, 137.5, 127.1, 119.1, 71.3, 62.7, 58.7, 57.0, 53.4, 38.6, 31.1, 29.4, 26.9, 19.3, 18.2.

These data correspond to those reported in the literature.[3]

4-((S)-2-((S)-2-(2-methoxyacetamido)-3-methylbutanamido)-5-ureidopentanamido)benzyl (4-methyl-2-oxo-2H-chromen-7-yl)carbamate (2e)

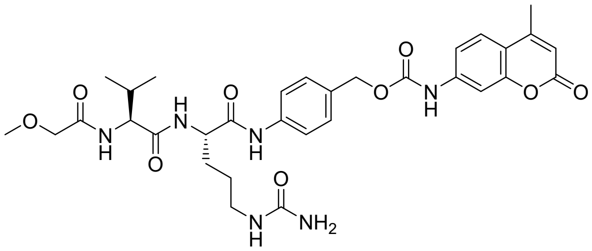

7-Amino-4-methylcoumarin (52.0 mg, 0.297 mmol) in CH_2Cl_2 (5 mL) was added triphosgene (15.0 mg, 50.5 µmol) then Et_3N (35.0 µL, 0.251 mmol). After 10 mins, (S)-*N*-(4-(hydroxymethyl)phenyl)-2-((S)-2-(2-methoxyacetamido)-3-methylbutanamido)-5-ureidopentanamide (**2d**) (52.0 mg, 0.115 mmol) in DMF (1 mL) was added. After stirring overnight, the reaction mixture was quenched with H_2O (0.5 mL), concentrated under a stream of N_2 and then purified by FCC (10-15% MeOH CH_2Cl_2) yielding 4-((*S*)-2-((*S*)-2-(2-methoxyacetamido)-3-methylbutanamido)-5-ureidopentanamido)benzyl (4-methyl-2-oxo-2H-chromen-7-yl)carbamate (**2e**) (46.0 mg, 70.5 µmol, 61%) as a white solid.

^{1}H NMR (500 MHz, DMSO-d$_6$): δ 10.27 (br s, 1H), 10.16 (br s, 1H), 8.36 (d, 1H, *J* = 7.5 Hz), 7.69 (d, 1H, *J* = 9.0 Hz), 7.61 (d, 2H, *J* = 8.4 Hz), 7.56 (d, 1H, *J* = 1.9 Hz), 7.47 (d, 1H, *J* = 8.8 Hz), 7.41 (dd, 1H, *J* = 8.9, 2.0 Hz), 7.38 (d, 2H, *J* = 8.6 Hz), 6.23 (d, 1H, J = 1.6 Hz), 6.08 (t, 1H, J = 5.9 Hz), 5.41 (br s, 2H), 5.12 (s, 2H), 4.40 (m, 1H), 4.30 (m, 1H), 3.86 (d, 2H, *J* = 1.2 Hz), 3.32 (s, 3H) 3.00-2.93 (m, 2H), 2.38 (d, 3H, J = 1.2 Hz), 2.00 (m, 1H), 1.70 (m, 1H), 1.59 (m, 1H), 1.44 (m, 1H), 1.36 (m, 1H), 0.86 (d, H, J = 6.9 Hz), 0.81 (d, 3H, J = 6.8 Hz); **^{13}C NMR** (101 MHz, DMSO-d$_6$): δ 170.8, 170.6, 168.7, 160.1, 158.9, 153.8, 153.2, 142.8, 139.0, 130.8, 129.2, 126.1, 119.0, 114.4, 114.3, 111.9, 104.4, 71.2, 66.0, 58.6, 56.7, 53.2, 38.4, 31.0, 29.2, 22.1, 19.2, 18.01, 18.00.

These data correspond to those reported in the literature.[3]

Non-cleavable Model Linker **3c**

Scheme S3: Synthesis of non-cleavable AMC model linker

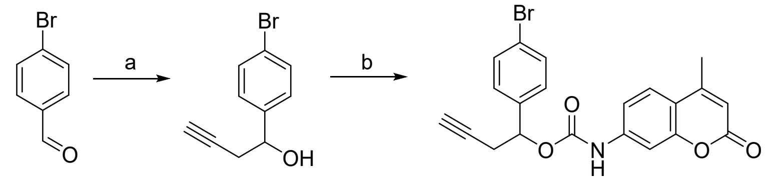

3b **3c**

Reagents and conditions: a) Activated Zn powder, diiodoethane, propargyl bromide, THF, sonication, rt, overnight, 68%; b) AMC, triphosgene, toluene, reflux 2 h, then **3b**, dibutyltin dilaurate, rt, 18 h, 52%.

1-(4-bromophenyl)but-3-yn-1-ol (3b)

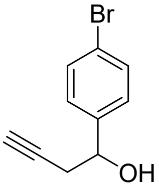

4-bromobenzaldehyde (500 mg, 2.70 mmol), activated Zn powder (882 mg, 13.5 mmol), diiodoethane (765 mg, 2.71 mmol) and propargyl bromide (80 wt% in toluene) (450 µL, 4.04 mmol) were suspended in THF (10 mL) and sonicated at rt for 2 h. 1N HCl (40 mL) was then added to the reaction mixture and was extracted with EtOAc (4 x 40 mL). The combined organic phases were washed with brine (40 mL), dried over Na_2SO_4 and concentrated *in vacuo*. The crude residue was purified by FCC (20% EtOAc in PE) yielding 1-(4-bromophenyl)but-3-yn-1-ol, **3b** (325 mg, 1.28 mmol, 68%) as a pale-yellow oil.

R_f = 0.16 (20% EtOAc in PE); **^{1}H NMR** (400 MHz, $CDCl_3$): δ 7.51 (d, 2H, *J* = 7.9 Hz), 7.29 (d, 2H, *J* = 7.6 Hz), 4.86 (t, 1H, *J* = 6.3 Hz), 2.63 (m, 2H), 2.10 (m, 1H); **^{13}C NMR** (101 MHz, $CDCl_3$): δ141.4, 131.6, 127.6, 121.8, 80.2, 71.7, 71.4, 29.4; **IR** ν_{max} /cm^{-1}: 3274br, 1724m, 1682s, 1615m, 1584m, 1533m; **HRMS (ESI)**: m/z calcd for [M+H]+ : 224.9910; found: 224.9920.

1-(4-bromophenyl)but-3-yn-1-yl (4-methyl-2-oxo-2H-chromen-7-yl)carbamate (3c)

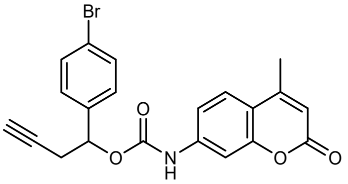

7-Amino-4-methylcoumarin (156 mg, 0.89 mmol) and triphosgene (131 mg, 0.44 mmol) were suspended in toluene (32 mL) and heated to reflux. After 2 h, the reaction mixture was cooled to rt and evaporated under a stream of nitrogen before a solution of 1-(4-bromophenyl)but-3-yn-1-ol, **3b** (200 mg, 0.89 mmol) in THF (32 mL) was added. Dibutyltin dilaurate (53.0 µL, 0.09 mmol) was added to the resulting suspension and stirred at rt for approx. 18 h before being quenched with H_2O (3 mL) and concentrated *in vacuo*. The crude residue was then purified by reverse-phase FCC (10-80% solvent B in solvent A. Solvent A: 0.5% formic acid (aq). Solvent B: MeCN), yielding 1-(4-bromophenyl)but-3-yn-1-yl (4-methyl-2-oxo-2H-chromen-7-yl)carbamate, **3c** (196 mg, 0.46 mmol, 52%) as a white solid.

^{1}H NMR (400 MHz, $CDCl_3$): δ 7.50-7.53 (m, 3H), 7.35-7.41 (m, 2H), 7.30-7.32 (m, 2H), 6.19 (br s, 1H), 5.87 (t, 1H, *J* = 6.5 Hz), 2.74-2.87 (m, 2H), 2.40 (br s, 3H), 2.03 (t, 1H, *J* = 2.7 Hz); **^{13}C NMR** (101 MHz, $CDCl_3$): δ 161.1, 154.5, 152.3, 151.8, 141.1, 137.7, 131.9, 128.3, 125.5, 123.0, 115.9, 114.5, 113.5, 106.2, 79.0, 74.4, 71.8, 26.5, 18.9; **IR** ν_{max} /cm^{-1}: 3274br, 1724m, 1682s, 1615m, 1584m; **HRMS (ESI)**: m/z calcd for [M+H]+ : 426.0342; found: 426.0343.

Unsubstituted DVP-Linker-MMAE **1f**

Scheme S4: Synthesis of Unsubstituted DVP-Linker-Payload 1f

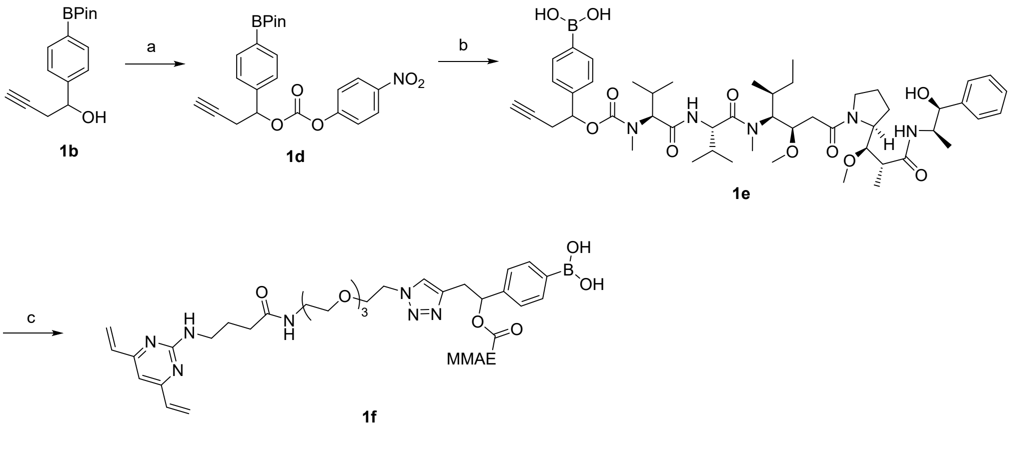

Reagents and conditions: a) *para*-nitrophenyl chloroformate, pyridine, CH_2Cl_2, 0 °C then rt, 3 h, 37%; b) MMAE, DMF, pyridine, DIPEA, HOBt, rt, overnight, 35%; c) **DVP-azide (5d)** CH_2Cl_2, $Cu_2SO_4{\cdot}5H_2O$, THPTA, sodium ascorbate, rt, 3.5 h, 23%.

4-nitrophenyl (1-(4-(4,4,5,5-tetramethyl-1,3,2-dioxaborolan-2-yl)phenyl)but-3-yn-1-yl) carbonate (1d)

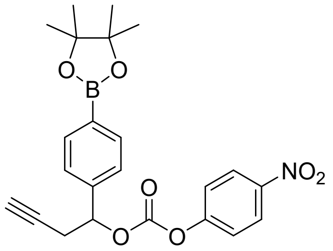

Para-nitrophenyl chloroformate (59.7 mg, 0.296 mmol), 1-(4-(4,4,5,5-tetramethyl-1,3,2-dioxaborolan-2-yl)phenyl)but-3-yn-1-ol (**1b**) (40.0 mg, 0.147 mmol), in CH_2Cl_2 (1 mL) were cooled to 0 °C before the addition of pyridine (17.0 µL, 0.211 mmol). The solution was then allowed to warm to rt over 3 h. The reaction mixture was then diluted with CH_2Cl_2 (10 mL) and extracted with sat. aq. $NaHCO_3$ (10 mL). The aqueous phase was then re-extracted with CH_2Cl_2 (2 x 10 mL). The combined organic phases were then dried over Na_2SO_4 then concentrated *in vacuo* before purifying by FCC (20-50% EA in PE), then a second FCC (20% EA in PE), yielding 4-nitrophenyl (1-(4-(4,4,5,5-tetramethyl-1,3,2-dioxaborolan-2-yl)phenyl)but-3-yn-1-yl) carbonate (**1d**) (26.0 mg, 0.055 mmol, 37%) as a colourless oil.

R_f = 0.21 (20% EtOAc in PE); **^{1}H NMR** (400 MHz, $CDCl_3$): δ 8.26 (d, 2H, *J* = 9.3 Hz), 7.86 (d, 2H, *J* = 8.2 Hz), 7.45 (d, 2H, *J* = 7.6 Hz), 7.36 (d, 2H, *J* = 9.3 Hz), 5.85 (t, 1H, *J* = 6.6 Hz), 2.80-2.97 (m, 2H), 1.35 (s, 12H); **^{13}C NMR** (101 MHz, $CDCl_3$): δ 155.6, 151.6, 145.5, 140.3, 135.2, 130.1, 125.8, 125.3, 121.8, 84.1, 79.0, 78.4, 71.4, 26.5, 24.9; **IR** ν_{max} /cm^{-1}: 3295w, 2978w, 2929w, 1765m, 1524m; **HRMS (ESI)**: m/z calcd for $[M]^-$: 438.1719; found 438.1715.

Unsubstituted-alkyne-MMAE (1e)

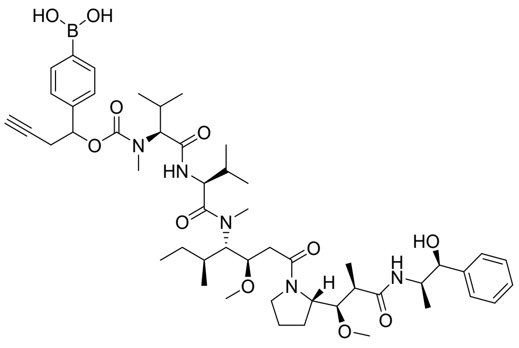

4-nitrophenyl (1-(4-(4,4,5,5-tetramethyl-1,3,2-dioxaborolan-2-yl)phenyl)but-3-yn-1-yl) carbonate **(1d)** (16.6 mg, 38.0 µmol) and MMAE (27.0 mg, 37.6 µmol) in DMF (1 mL) were added pyridine (82.5 µL, 1.33 mmol), DIPEA (6.60 µL, 37.9 µmol) and HOBt (80 wt%) (7.00 mg, 41.5 µmol). After stirring overnight at rt, the reaction mixture was concentrated *in vacuo* then purified by reverse-phase FCC (10-80% solvent B in solvent A. Solvent A: H_2O (aq). Solvent B: MeCN). Any boronic ester product was subjected to hydrolysis in H_2O:NH_4OH (aq), stirred at rt overnight, then lyophilised yielding Unsubstituted-alkyne-MMAE **(1e)** (12.5 mg, 13.4 µmol, 35%) as a white powder.

HPLC (5-95% MeCN/H_2O over 20 min) retention time 11.847 min; **HPLC** (20-80% MeCN/H_2O over 20 min) retention time 11.325 min; **LRMS** (ESI) m/z found [M+H]+ : 934.7, required 934.9.

Unsubstituted-DVP-MMAE (1f)

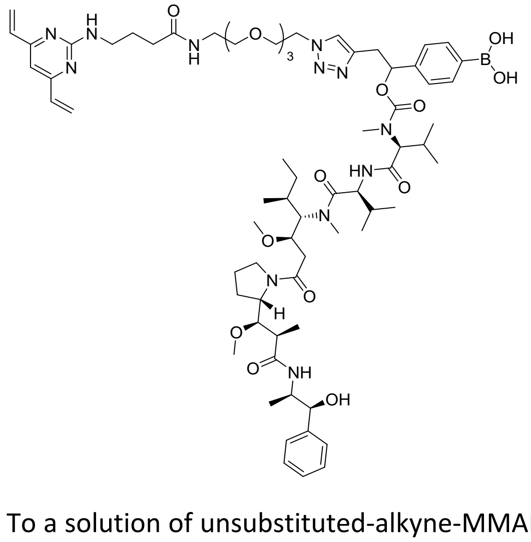

To a solution of unsubstituted-alkyne-MMAE **(1e)** (12.5 mg, 13.4 µmol) and DVP-azide **(5d)** (12.9 mg, 29.8 µmol) in CH_2Cl_2 (1.8 mL), was added a solution of $CuSO_4 \cdot 5H_2O$ (4.0 mg, 16.0 µmol), THPTA (11.5 mg, 26.5 µmol) and sodium ascorbate (13.3 mg, 67.1 µmol) in $H_2O/^tBuOH$ (3.6 mL, 1:1) and the reaction mixture was stirred at rt for 3.5 h. The reaction mixture was then concentrated *in vacuo* and purified by reverse-phase FCC (10-80% solvent B in solvent A. Solvent A: 0.1 M NH_4OH (aq). Solvent B: MeCN). Lyophilisation yielded Unsubstituted-DVP-MMAE **(1f)** (4.20 mg, 3.07 µmol, 23%) as a white powder.

HPLC (5-95% MeCN/H_2O over 20 min) retention time 9.831 min; **HPLC** (20-80% MeCN/H_2O over 20 min) retention time 9.158 min; **HRMS (ESI)**: m/z calcd for [M+H]+: 1366.8162; found 1366.8156.

Non-cleavable DVP-Linker-MMAE was provided by Stephen Walsh.

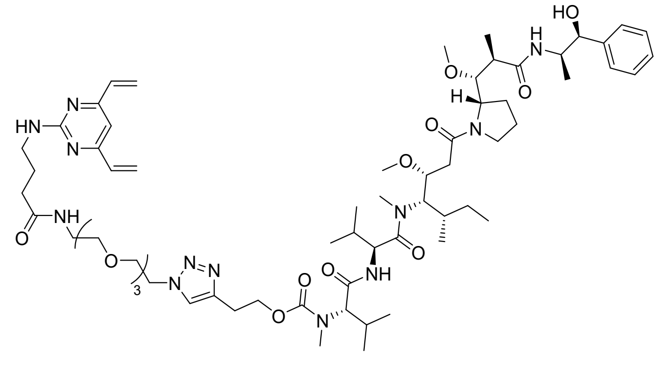

Non-cleavable DVP-Linker-MMAE

Val-Cit DVP-Linker-MMAE **2f** was provided by Stephen Walsh.

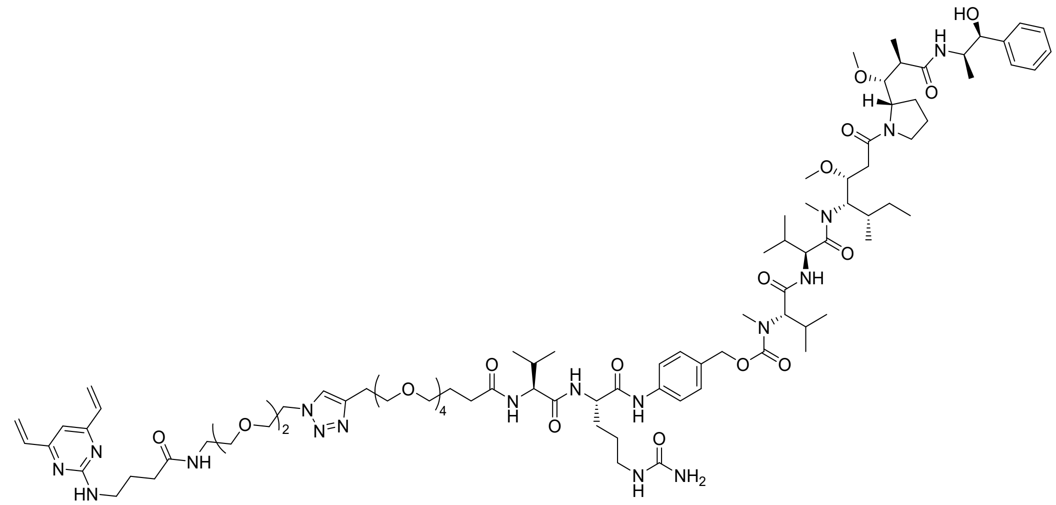

Val-Cit DVP-Linker-MMAE

DVP-Azide **5d** was provided by Stephen Walsh.

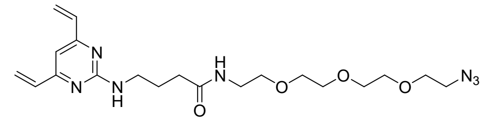

5d

Experimental References:

[1] R. Bisht, B. Chattopadhyay, *J. Am. Chem. Soc.* **2016**, *138*, 84–87.

[2] D. Mondal, J. Ford, K. G. Pinney, *Tetrahedron Lett.* **2018**, *59*, 3594–3599.

[3] J. D. Bargh, S. J. Walsh, A. Isidro-Llobet, S. Omarjee, J. S. Carroll, D. R. Spring, *Chem. Sci.* **2020**, *11*, 2375–2380.

Bioconjugation

To a solution of trastuzumab or durvalumab (40 µL, 16.9 µM, 2.5 mg/mL) in tris-buffered saline (TBS) (25 mM Tris HCl pH 8, 25 mM NaCl, 0.5 mM EDTA) was added TCEP (10 eq.). The mixture was vortexed and incubated at 37 °C for 1 h with shaking at 400 rpm. A solution of DVP-linker-payload (20 mM in DMSO) was added with additional DMSO (final concentration of 0.61 mM, for 40 eq of linker or 1.22 mM for 80 eq of linker, 10% DMSO (v/v)) and the reaction mixture incubated at 37 °C for 4 h with shaking at 400 rpm. The excess reagents were removed by size-exclusion chromatography with a Zeba Spin desalting column (40K MWCO, 0.5 mL) and exchanged into PBS with an Amicon-Ultra centrifugal filter (10K MWCO, Merck Millipore).

Table SI-1: Summary of bioconjugation reaction conditions of trastuzumab and durvalumab to generate ADCs 1-4 and 5-7 respectively.

ADC	Antibody	Linker	Linker equivalents	Reaction time	Average DAR
**1**	Trastuzumab	DVP-unsubstituted-MMAE, **1f**	40	4 h	3.7
**2**	Trastuzumab	DVP-Val-Cit-MMAE	80	4 h	3.9
**3**	Trastuzumab	DVP-non-cleavable-MMAE	80	4 h	3.8
**4**	Durvalumab	DVP-unsubstituted-MMAE, **1f**	40	4 h	3.6
**5**	Durvalumab	DVP-non-cleavable-MMAE	40	4 h	3.7
**6**	Durvalumab	DVP-Val-Cit-MMAE	40	4 h	3.7

SDS-PAGE

Non-reducing Tris-Glycine SDS-PAGE with12% acrylamide with 4% stacking gel was performed as standard. Broad range molecular weight marker (10-200 kDa, New England BioLabs) was run in all gels. Samples (5 µL, 2.5 µg unless stated otherwise) were prepared with reducing loading dye (5 µL, containing β-mercaptoethanol) and heated to 90 °C for 5 min before loading. Gels were run at constant voltage (200 V) for 45-60 min in 1 x Laemmli running buffer (LRB). All gels were stained with Coomassie blue dye and imaged on a Syngene gel imaging system.

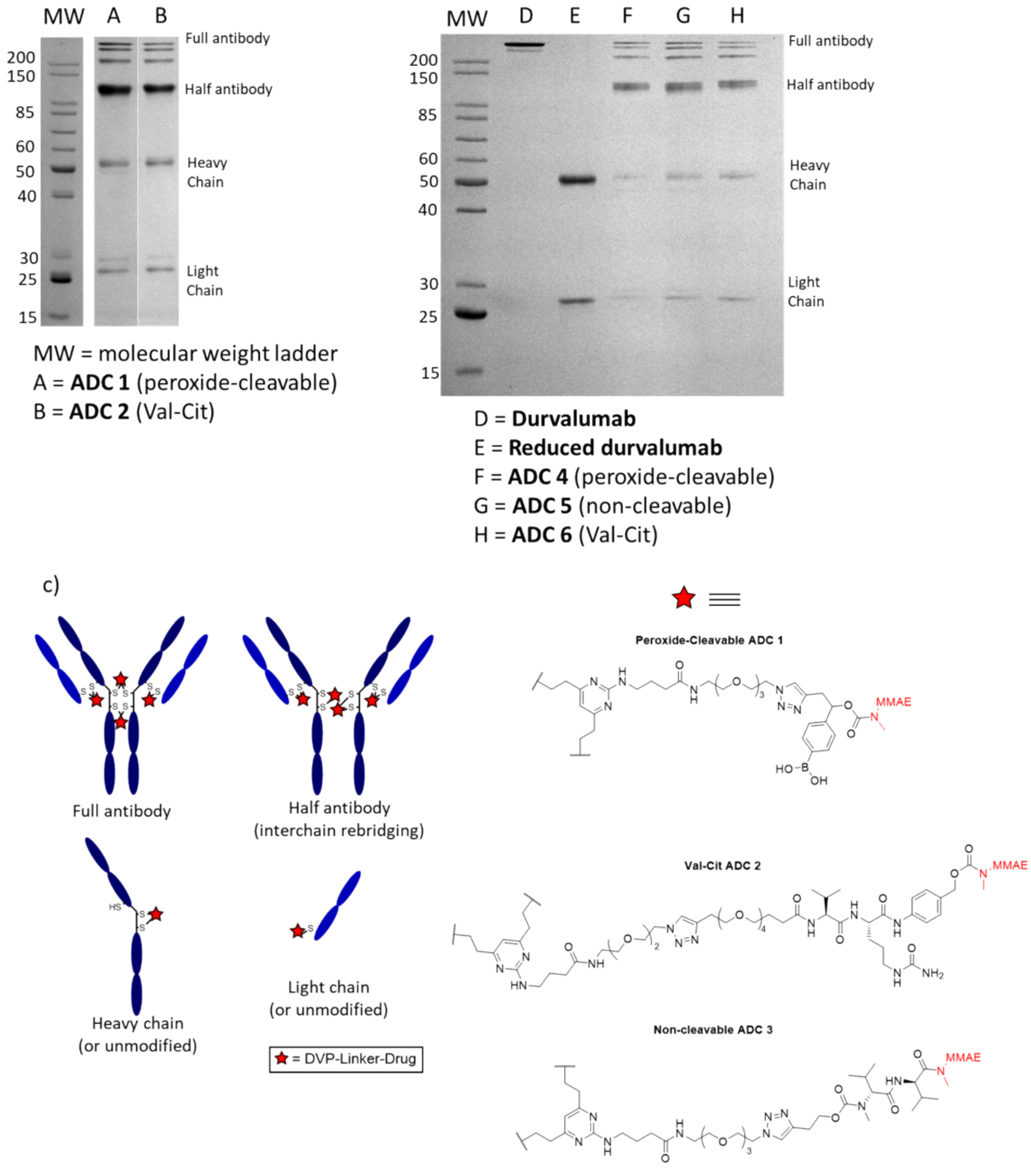

Figure S1: Reducing SDS-page gel for the bioconjugation reactions a) to form **ADC 1** (from **1f**), **ADC 2** (from **2f**) and b) to form **ADCs 4-6.** The molecular weight ladder is shown to the left of the lanes ran on the same gel and images have been cropped to remove irrelevant lanes. c) representations of the different antibody species present on the gel and structure of each linker. SDS-PAGE analysis

demonstrates that the major product of bioconjugation is the half-antibody species, which has a molecular weight between that of the full trastuzumab/durvalumab antibody and the heavy chain.

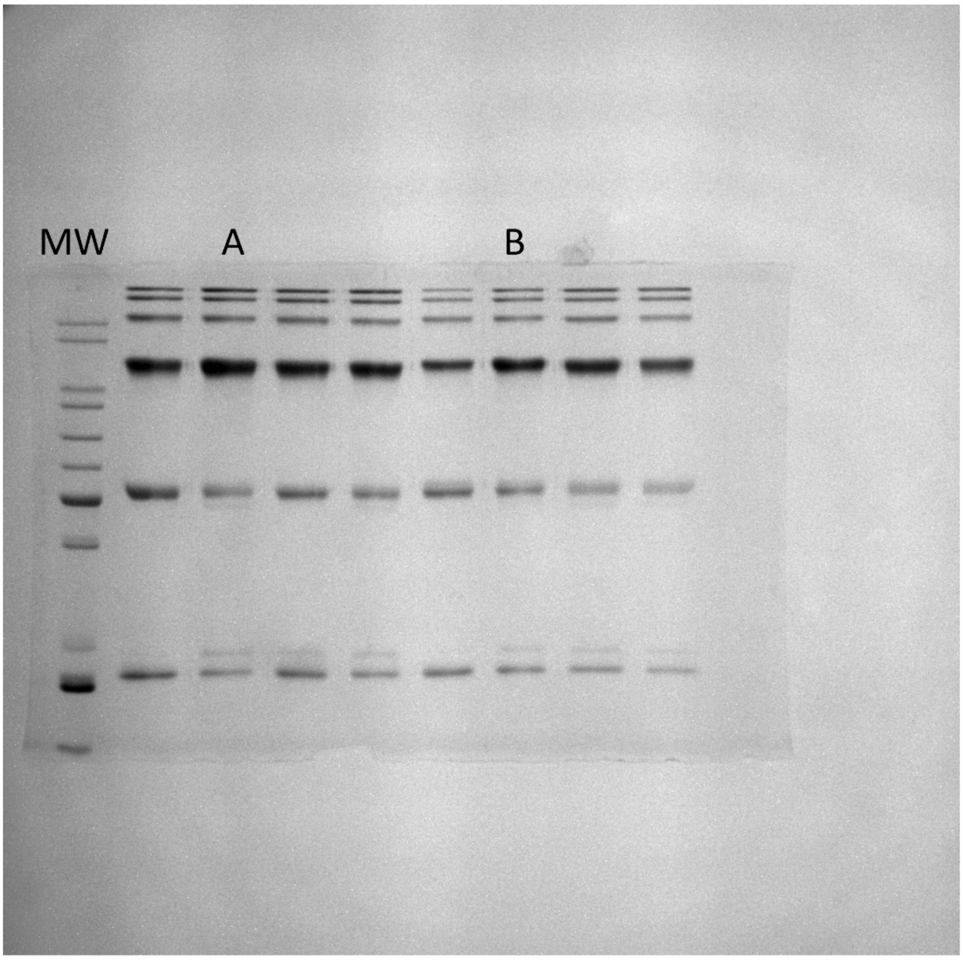

Figure S1-a: Raw, uncropped, un-processed SDS-Page Gel bioconjugation reactions a) to form **ADC 1** (from **1f**), **ADC 2** (from **2f**). Lanes not labelled in this raw image were not of relevance to this publication.

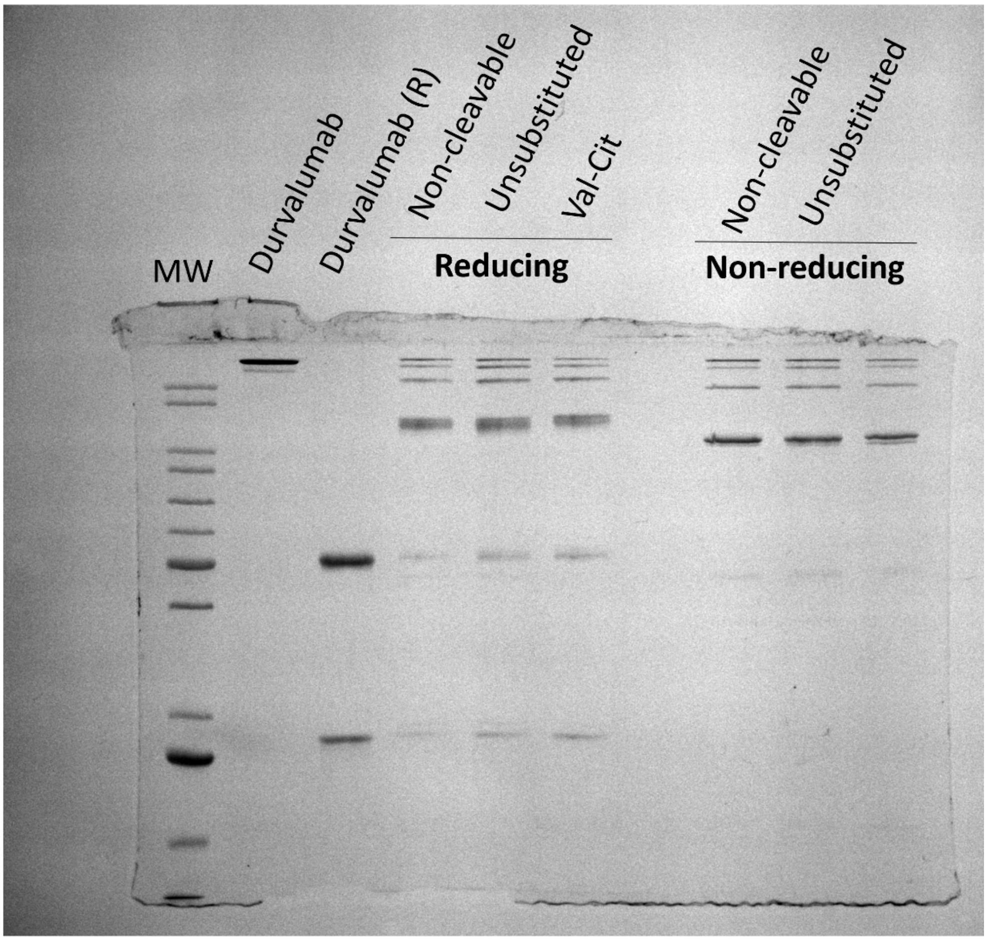

Figure S1-b: Raw, uncropped SDS-Page gel for the bioconjugation reactions to form **ADCs 4-6**.

Size-Exclusion Chromatography

Analytical size-exclusion chromatography (SEC) was carried out on an AKTA pure chromatography system using a Superdex™ 200 Increase 10/300 GL column. Samples were injected at a concentration of 1 mg/mL and eluted with TBS pH 8 (25 mM Tris HCl, 200 mM NaCl, 0.5 mM EDTA) at a flow rate of 0.5 mL/min.

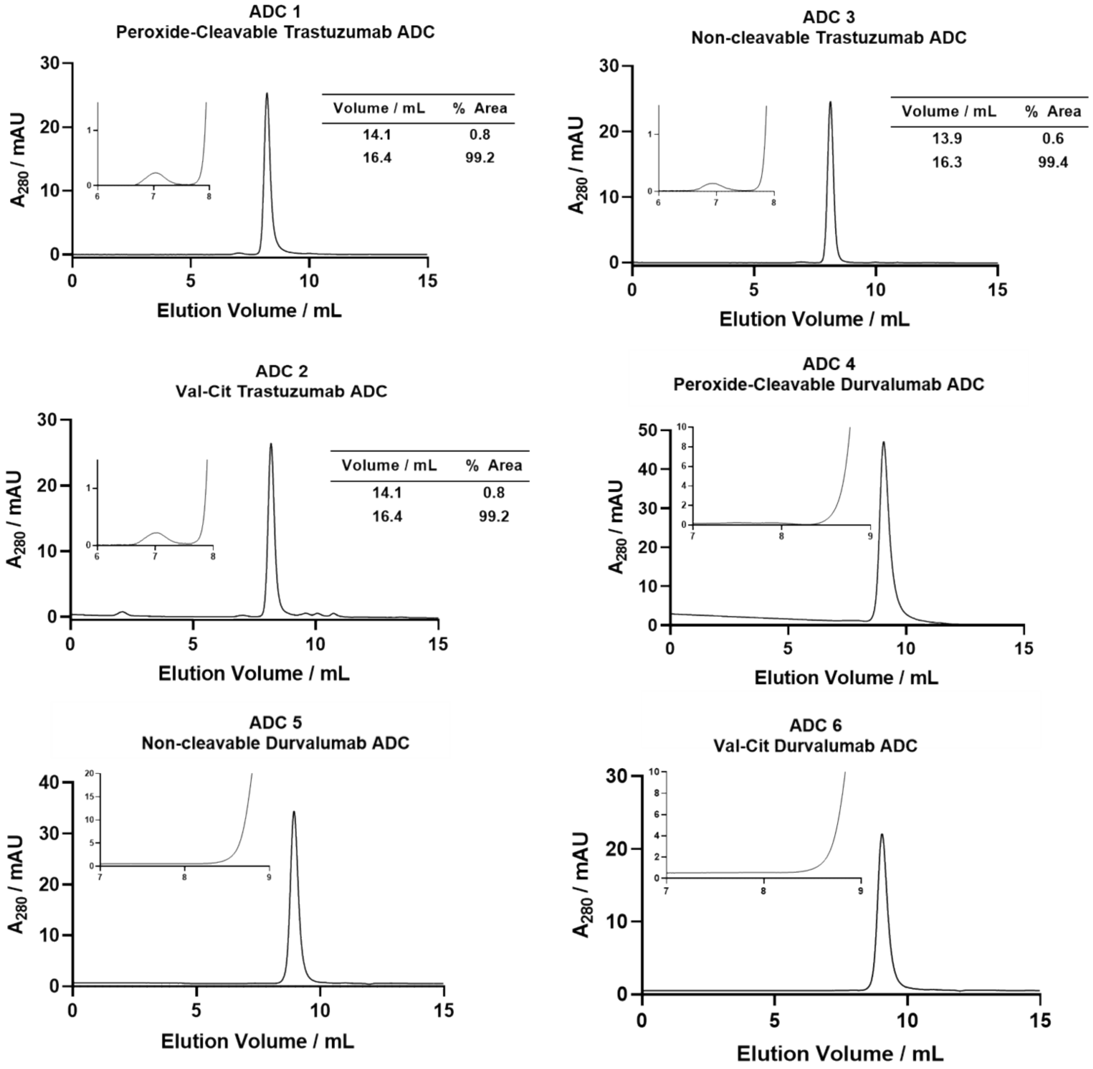

Figure S2: Size-exclusion chromatography of **ADCs 1, 2 & 3**, highlighting the low levels of ADC aggregation (< 1%). No aggregation was observed for **ADCs 4-6**.

Hydrophobic Interaction Chromatography

Analytical hydrophobic interaction chromatography (HIC) was carried out on a Tosoh Bioscience TSKgel Butyl-NPR column (3.5 cm × 4.6 mm, 2.5 μm). Samples were injected at a concentration of 1 mg/mL and eluted with a linear gradient of solvent A in solvent B (solvent A: 1.5 M ammonium sulfate, 25 mM NaPi, pH 7 and solvent B: 25% isopropyl alcohol in 25 mM NaPi, pH 7 at a flow rate of 0.6 mL/min. The drug-to-antibody ratio was calculated by the integration of the signals at 280 nm:

$$Average\ DAR = \frac{(DAR_1 + 2 \times DAR_2 + 3 \times DAR_3 + 4 \times DAR_4 + 5 \times DAR_5)}{(DAR_0 + DAR_1 + DAR_2 + DAR_3 + DAR_4 + DAR_5)}$$

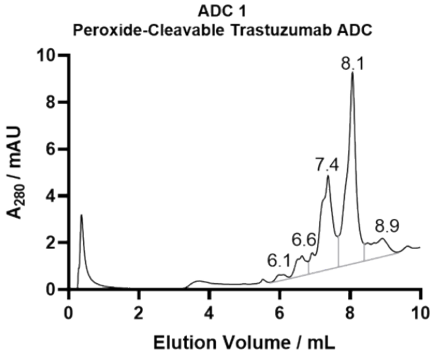

Peak Retention / mL	% Area
6.1	1.4
6.6	4.7
7.4	26.6
8.1	45.6
8.9	7.5

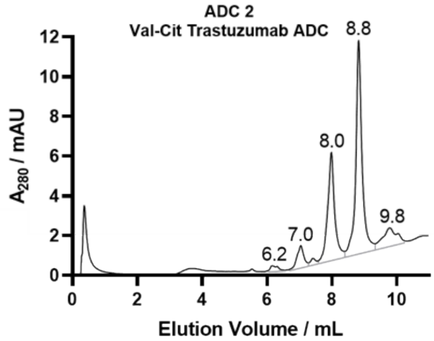

Peak Retention / mL	% Area
6.2	1.6
7.0	5.0
8.0	26.4
8.8	46.7
9.8	7.2

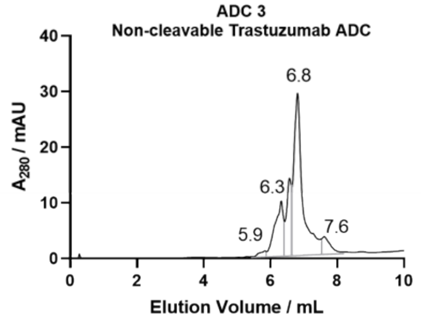

Peak Retention / mL	% Area
5.9	1.7
6.3	20.7
6.8	53.5
7.6	6.0

Figure S3: Hydrophobic interaction chromatography of the product of bioconjugation reactions of trastuzumab to form **ADCs 1-3.** Graphs are annotated with the elution volume (mL) and the grey lines represent integrations of the curve used to calculate average drug-to-antibody ratio.

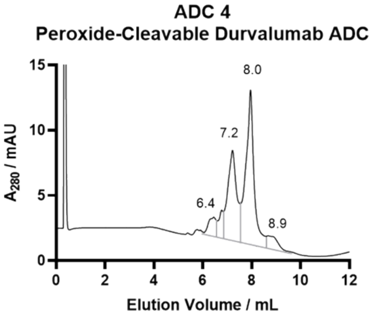

Peak Retention / mL	% Area
6.4	7.2
7.2	31.9
8.0	50.2
8.9	5.6

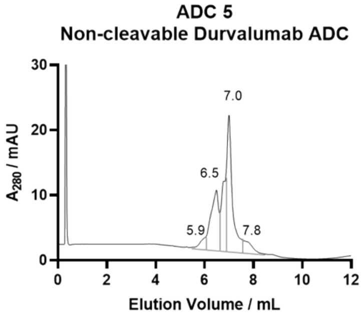

Peak Retention / mL	% Area
5.9	4.2
6.5	26.2
7.0	43.7
7.8	7.7

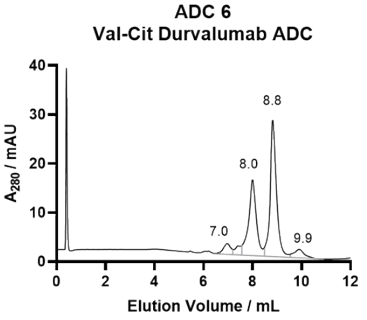

Peak Retention / mL	% Area
7.0	5.5
8.0	34.4
8.8	50.9
9.9	6.0

Figure S4: Hydrophobic interaction chromatography of the product of bioconjugation reactions of durvalumab to form **ADCs 4-6.** Graphs are annotated with the elution volume (mL) and the grey lines represent integrations of the curve used to calculate average drug-to-antibody ratio.

Protein LC-MS

Protein LC–MS was performed on a Xevo G2-S TOF mass spectrometer coupled to an Acquity UPLC system using an Acquity UPLC BEH300 C4 column (1.7 µm, 2.1 × 50 mm). H_2O with 0.1% formic acid (solvent A) and 95% MeCN and 5% H_2O with 0.1% formic acid (solvent B) were used as the mobile phase at a flow rate of 0.2 mL/min. The gradient was programmed as follows: 95% A for 0.93 min, then a gradient to 100% B over 4.28 min, then 100% B for 1.04 minutes, then a gradient to 95% A over 1.04 min. The electrospray source was operated with a capillary voltage of 2.0 kV and a cone voltage of 190 V, 150V or 40 V. Nitrogen was used as the desolvation gas at a total flow rate of 850 L/h. Total mass spectra were reconstructed from the ion series using the MaxEnt 1 algorithm preinstalled on MassLynx 4.2 software according to the manufacturer's instructions. Trastuzumab samples were deglycosylated with PNGase F (New England Biolabs) prior to LC-MS analysis. Only the region of the total ion chromatogram (TIC) between 3.25-3.75 min was analysed. Peaks outside of this range did not contain proteinogenic signals and were excluded. Analysis was conducted in the same way for all protein LC-MS traces.

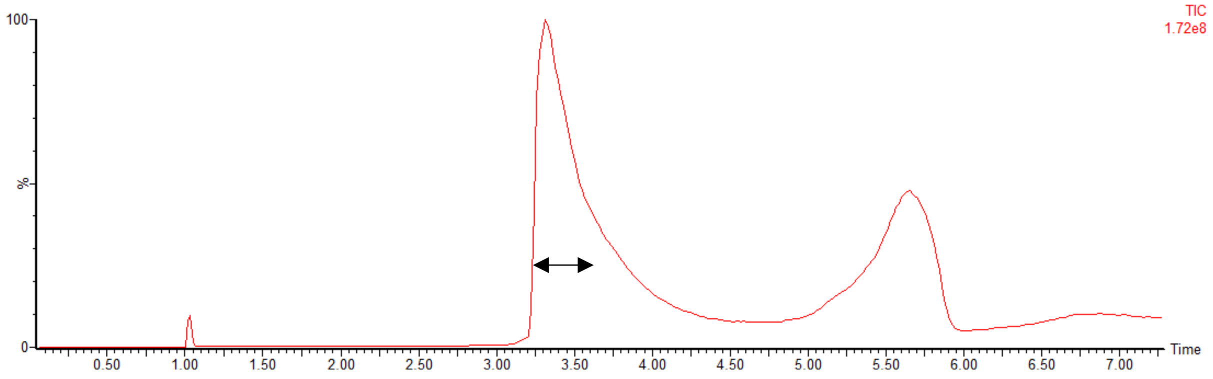

Figure S5: Typical TIC trace of trastuzumab sample with arrow indicating region of analysis.

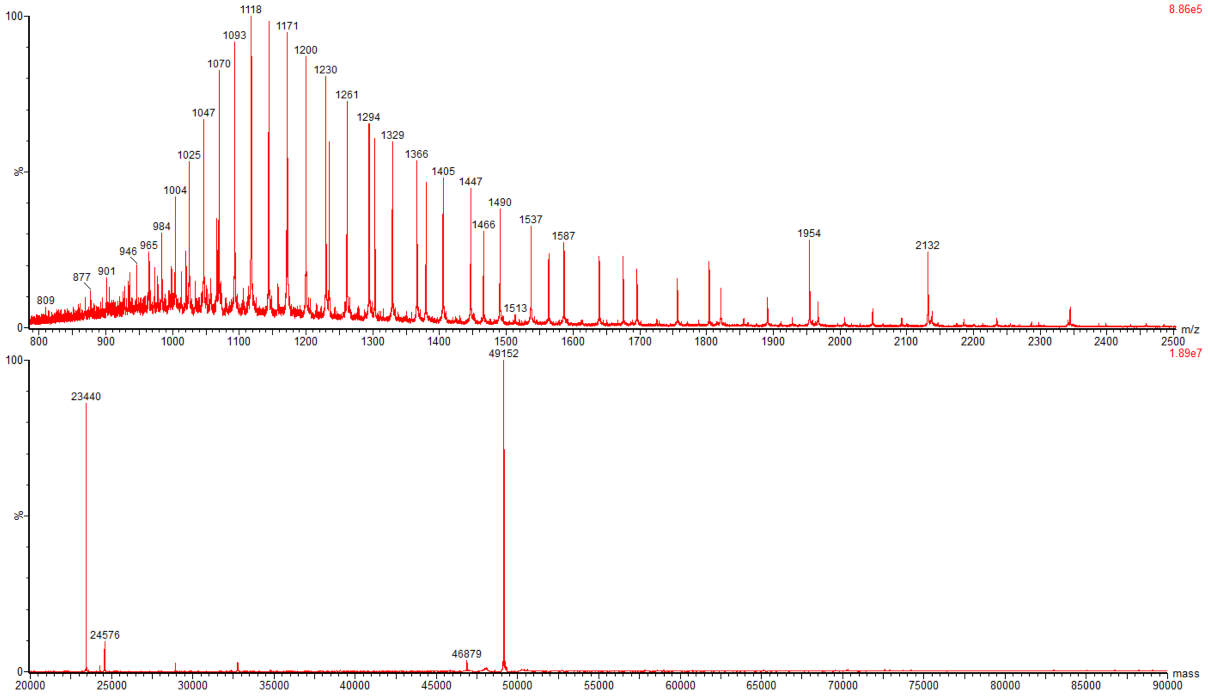

Figure S6: LC-MS of unmodified, deglycosylated, reduced trastuzumab. Top = non-deconvoluted MS. Bottom = deconvoluted MS; heavy chain expected 49,153 Da, observed 49,152 Da; light chain expected 23,439 Da, observed 23,440 Da.

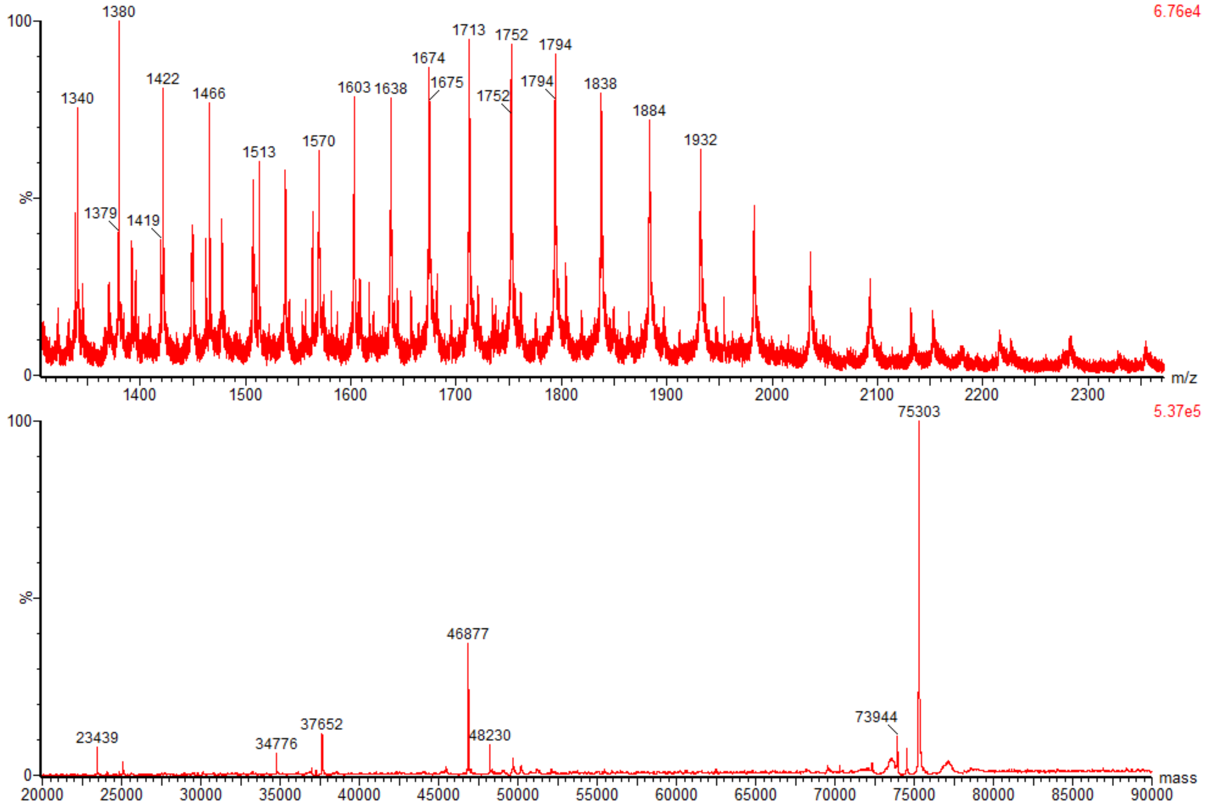

Figure S7: LC-MS of **ADC 1**. Top = non-deconvoluted MS. Bottom = deconvoluted MS; expected 75,327 Da, observed 75,303 Da.

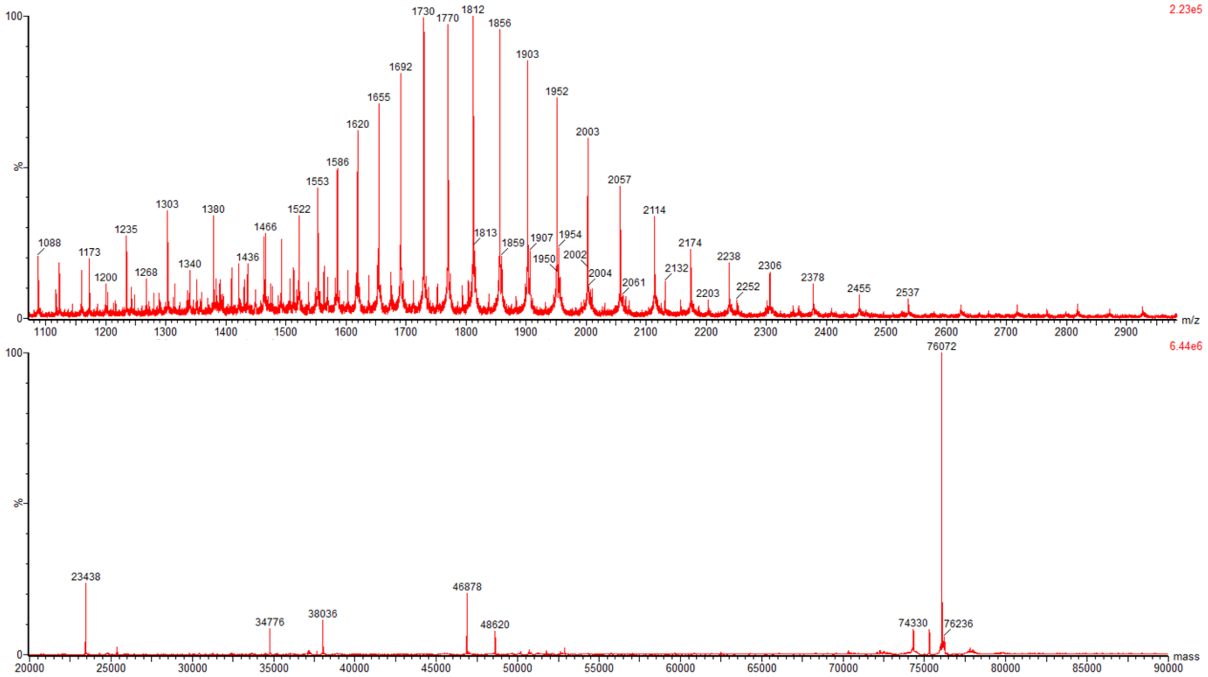

Figure S8: LC-MS of **ADC 2**. Top = non-deconvoluted MS. Bottom = deconvoluted MS; expected 76,074 Da, observed 76,072 Da.

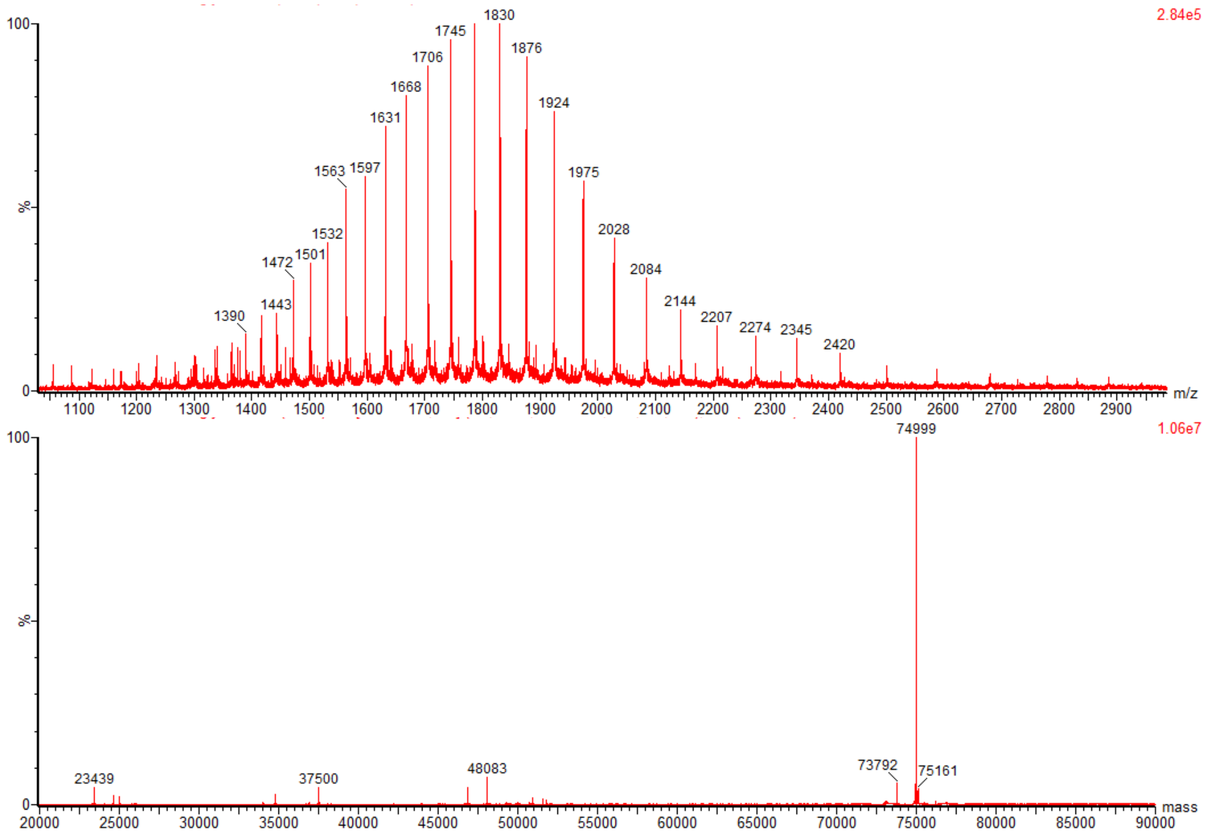

Figure S9: LC-MS of **ADC 3**. Top = non-deconvoluted MS. Bottom = deconvoluted MS; expected 74,998 Da, observed 74,999 Da.

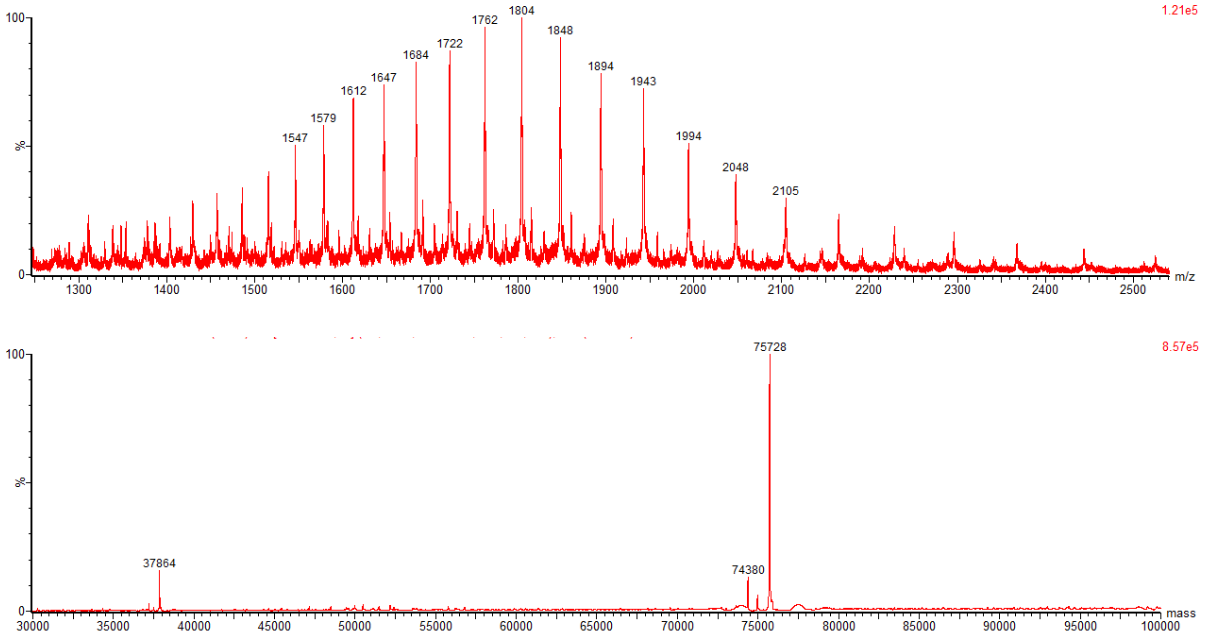

Figure S10: LC-MS of **ADC 4**. Top = non-deconvoluted MS. Bottom = deconvoluted MS; expected 75,774 Da, observed 75,728 Da.

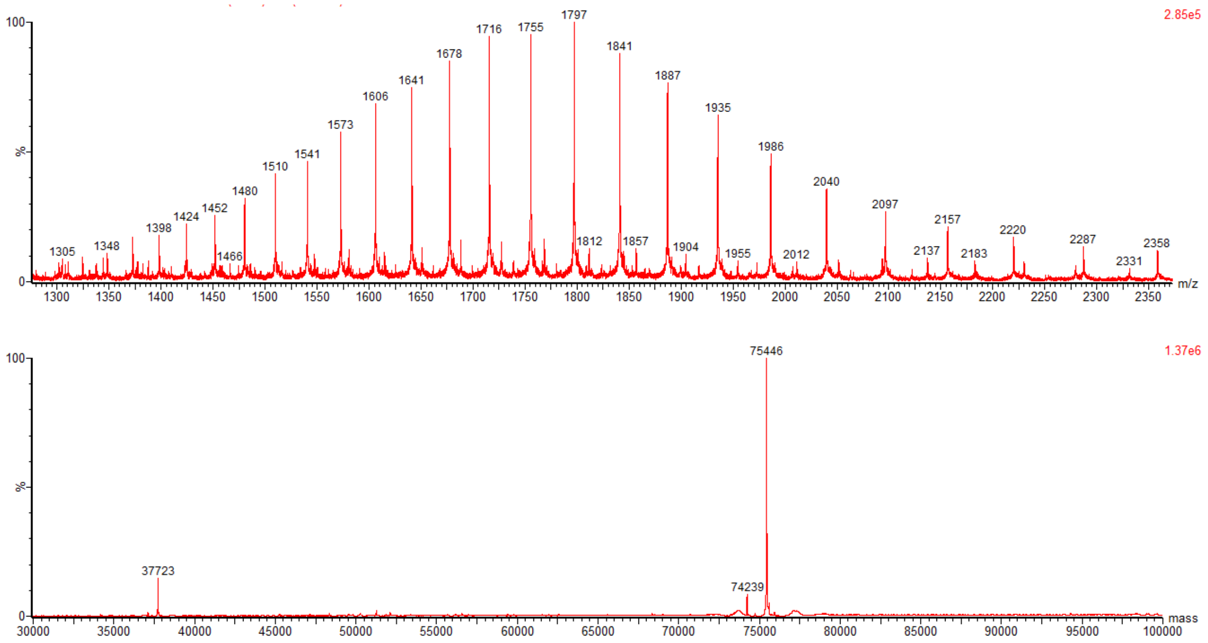

Figure S11: LC-MS of **ADC 5**. Top = non-deconvoluted MS. Bottom = deconvoluted MS; expected 75,444 Da, observed 75,446 Da.

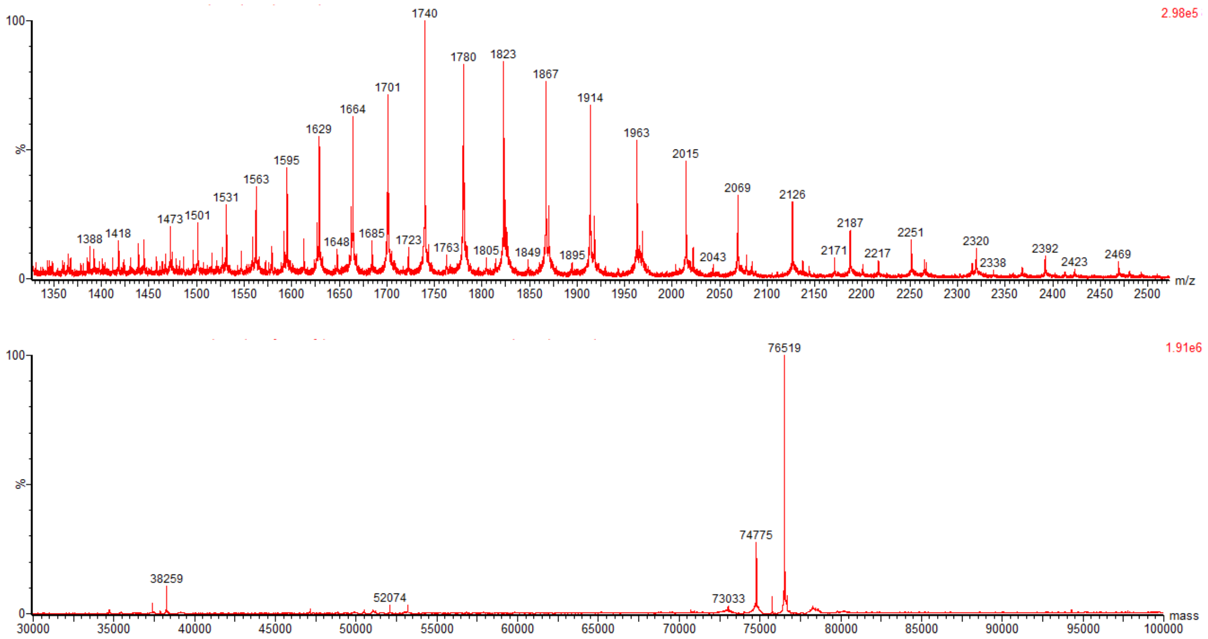

Figure S12: LC-MS of **ADC 6**. Top = non-deconvoluted MS. Bottom = deconvoluted MS; expected 76,520 Da, observed 76,519 Da.

Peroxide-Cleavage Studies

Data from all cleavage studies were processed using GraphPad Prism Version 9.3.0.
Human and mouse plasma were obtained from Sigma Aldrich.

Release from model linkers **1c**, **2e** and **3b** with 0, 1, 5, 10 equivalents of hydrogen peroxide

Model linkers **1c**, **2e**, **3b** (7.2 µL, 2.5 mM DMSO) were vortexed with PBS (280.8 µL) before adding 72.0 µL of hydrogen peroxide solution (1, 5, 10 equivalents – final concentration of 50 µM, 250 µM and 500 µM respectively). 340 µL of the resulting solution was added to a 96-well plate (Greiner, black, clear flat bottomed). Fluorescence intensity was monitored at λ_{abs} = 441 nm over 20 h at 37 °C. An adhesive film (BioRad) was used to prevent solvent evaporation. Readings were taken at 30-35 second intervals. The reactions were performed in triplicate, with three technical replicates for each set of conditions. Control wells contained no hydrogen peroxide but 72.0 µL of water instead.

Release in the presence of hydrogen peroxide scavenger CAT

Model linkers **1c** (7.2 µL, 2.5 mM DMSO) was vortexed with either PBS (280.8 µL) containing ~2000 U/mL catalase (CAT) from bovine liver. 72.0 µL hydrogen peroxide solution (10 equivalents) (or 72.0 µL water for control wells) was then added to the solutions and 340 µL of the resulting solution was added to a 96-well plate (Greiner, black, clear flat bottomed). Fluorescence intensity was monitored at λ_{abs} = 441 nm over 25 h at 37 °C. An adhesive film (BioRad) was used to prevent solvent evaporation. Readings were taken at 1.5-minute intervals. The reactions were performed in triplicate with three technical replicates for each set of conditions. Catalase was obtained from MERCK LIFE SCIENCE UK LTD.

Long term release in the presence of CAT scavenger

Once complete, the plate containing scavenger CAT was covered in foil and placed in an incubator at 37 °C for 7 days. Additional fluorimetry measurements were taken at t = 24, 48, 72, 120 and 240 h. This was performed in duplicate, with three technical replicates of each condition.

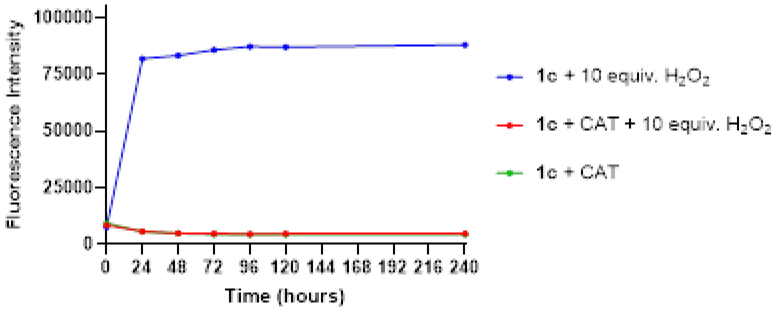

Figure S13: The analysis of fluorescence release from **1c** in the presence of 10 equivalents hydrogen peroxide and ROS scavenger Catalase (~2000 U/mL) over 10 days.

Stability Studies

<u>Stability of **1c** over 20 hours in human and mouse plasma by fluorimetry</u>

Model linker **1c** (36.0 µL, 2.5 mM DMSO) were vortexed with PBS (774 µL) and 153 µL of the resulting solution was added to a 96-well plate (Greiner, black, clear flat bottomed). Then, 187 µL human plasma was added to each well. Fluorescence intensity was monitored at λ_{abs} = 441 nm over 20 h at 37 °C. An adhesive film (BioRad) was used to prevent solvent evaporation. Readings were taken at 30-35 second intervals. The reactions were performed in triplicate, with three technical replicates for each set of conditions.

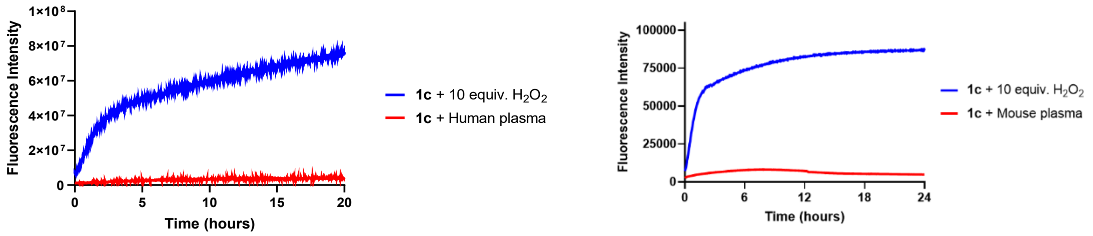

Figure S14: The fluorescence intensity of **1c** when incubated with human or mouse plasma. Also shown is **1c** with 10 equivalents of hydrogen peroxide for comparison.

<u>Stability of **1c** vs **Val-Cit 2e** in human and mouse plasma over 10 days (HPLC)</u>

1c and **4e** (10 mM) were incubated with PBS (149 µL), human/mouse plasma (265 µL), DMSO (86 µL) and caffeine (10 µL, 15 mg/mL, internal standard) at 37 °C for 10 days. 50 µL aliquots of the mixture were taken at t = 1, 2, 3, 4, 5, 10 days and flash frozen in liquid nitrogen and stored at -20 °C before HPLC analysis of all samples on the same day. The linker peak area was calibrated to the caffeine internal standard for quantification of degradation over time. One representative dataset is shown of two independent replicates.

Table SI-2: 2e Val-Cit model linker HPLC assay stability in human plasma.

Time (days)	Linker Peak Area	Caffeine Peak Area	Linker/ Caffeine	Normalised Linker %
**0**	193.9	1605.6	0.121	100
**1**	189.6	1581.1	0.120	99
**2**	184.3	1628.6	0.113	94
**3**	181.2	1493.4	0.121	100
**4**	195.2	1625.4	0.120	99
**5**	200.6	1729.7	0.116	96
**10**	180.6	1661.7	0.109	90

Table SI-3: 2e Val-Cit model linker HPLC assay stability in mouse plasma

Time (days)	Linker Peak Area	Caffeine Peak Area	Linker/ Caffeine	Normalised Linker %
**0**	185.1	1548.1	0.120	100
**1**	115.9	1564.4	0.074	62
**2**	65.9	1547.8	0.043	36
**3**	48.7	1658.5	0.029	25
**4**	29.0	1578.0	0.018	15
**5**	25.1	1547.3	0.016	14
**10**	0.0	1873.3	0.000	0

Table SI-4: 1c unsubstituted model linker HPLC assay stability in human plasma

Time (days)	Linker Peak Area	Caffeine Peak Area	Linker/ Caffeine	Normalised Linker %
**0**	421.2	1075.9	0.391	100
**1**	783.4	2299.7	0.341	87
**2**	662.2	2205.9	0.300	77
**3**	1009.5	3215.4	0.314	80
**4**	672.4	2111.7	0.318	81
**5**	608.3	2022.6	0.301	77
**10**	517.7	1989.2	0.260	66

Table SI-5: 1c unsubstituted model linker HPLC assay stability in mouse plasma

Time (days)	Linker Peak Area	Caffeine Peak Area	Linker/ Caffeine	Normalised Linker %
**0**	1317.7	1743.6	0.756	100
**1**	1330.4	1607.6	0.828	110
**2**	1486.9	1712.3	0.868	115
**3**	1321.4	1634.9	0.808	107
**4**	1394.4	1649.7	0.845	112
**5**	1193.8	1537.1	0.777	103
**10**	1335.6	1646.1	0.811	107

Stability of **1c** vs **Val-Cit** **2e** in human and mouse plasma over 10 days (fluorimetry)

Linkers **1c** and **2e** (28 µL, 2.5 mM in DMSO) were vortexed with PBS (602 µL) and 90 µL of this solution was added to the

wells of a 96-well plate (Greiner, black, clear flat bottomed). To this, plasma (from human or mouse, 110 µL) was added. An adhesive film (BioRad) was used to prevent solvent evaporation. Control wells replaced plasma with PBS. Each condition had three technical replicates. The plate was incubated at 37 °C for 10 days, with readings at λ_{abs} = 441 nm at t = 1, 2, 3, 4, 5, 7, 10 days.

The decrease in fluorescence of **2e** over time is thought to be due to photo-bleaching of the released fluorophore.

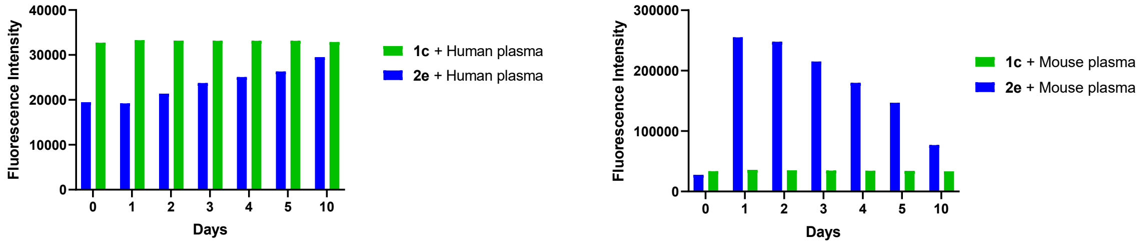

Fig S15: The fluorescence intensity of **1c** and **4e** when incubated with human (left) or mouse plasma (right) for 10 days.

Stability of **1c** in unconditioned cell media

Unconditioned cell media used had not been in contact with cancer cells. McCoy's = High glucose McCoy's 5A medium, supplemented with 10% heat-inactivated foetal-bovine serum (FBS), GlutaMAX™, 50 U/mL penicillin and 50 µg/mL streptomycin. RPMI = RPMI1640 medium supplemented with 10% FBS, 2 mM L-glutamine, 50 U/mL penicillin and 50 µg/mL streptomycin. DMEM = Dulbecco's Modified Eagle Medium (DMEM) supplemented with 10% FBS, 2 mM L-glutamine, 50 U/mL penicillin and 50 µg/mL streptomycin.

Model linker **1c** (7.2 µL, 2.5 mM DMSO) was vortexed with unconditioned cell media (280.8 µL) and then 272 µL was added to a 96-well plate (Greiner, black, clear flat bottomed). Water or hydrogen peroxide solution (10 equiv.) (68 µL) was then added to the appropriate wells and absorbance was monitored at λ_{abs} = 441 nm over 24 h at 37 °C. An adhesive film (BioRad) was used to prevent solvent evaporation. The reactions were performed in triplicate, with three technical replicates for each set of conditions.

Long term release in the presence of unconditioned cell media

Once complete, the plate containing **1c** in unconditioned cell media was covered in foil and placed in an incubator at 37 °C for the remaining 3 days. Additional fluorimetry measurements were taken at t = 48, 72 and 96 h. This was performed in triplicate, with three technical replicates of each condition.

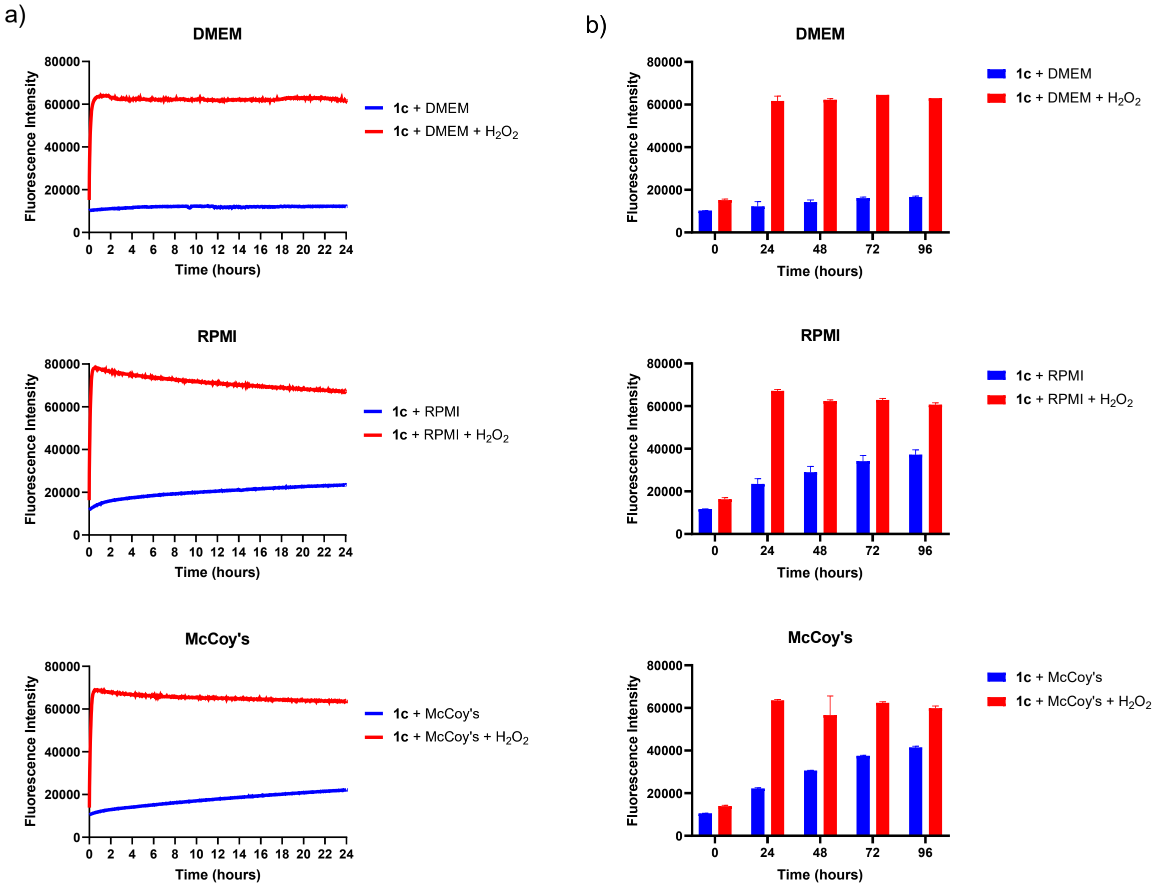

Figure S16: The fluorescence intensity of **1c** when incubated at 37 °C with unconditioned media with and without hydrogen peroxide (10 equiv.) for (a) 24 h, then (b) over 4 days.

Cell Lines

HER2-positive SKBR3 and BT474 cells were obtained from the American Type Culture Collection (ATCC) and HER2-negative MCF7 cells were obtained from the European Collection of Authenticated Cell Cultures (ECACC) and ATCC, respectively. MDA-MB-231 cells were obtained from ATCC. SKBR3 cells were maintained in high glucose McCoy's 5A medium, supplemented with 10% heat-inactivated foetal-bovine serum (FBS), GlutaMAX™, 50 U/mL penicillin and 50 µg/mL streptomycin. MCF7 cells were maintained in Dulbecco's Modified Eagle Medium (DMEM) supplemented with 10% FBS, 2 mM L-glutamine, 50 U/mL penicillin and 50 µg/mL streptomycin. BT474 and MDA-MB-231 cells were maintained in RPMI1640 medium supplemented with 10% FBS, 2 mM L-glutamine, 50 U/mL penicillin and 50 µg/mL streptomycin. All cell lines were incubated at 37 °C with 5% CO_2.

In Vitro Cytotoxicity

General Cell Viability Protocol

Cells were seeded in 96-well plates for 24 h at 37 °C with 5% CO_2. SKBR3 cells were seeded at 15,000 cells/well, BT474 cells were seeded at 20,000 cells/well, MCF7 cells were seeded at 7,500 cells/well and MDA-MB-231 cells were seeded at 5000 cells/well. Serial dilutions of ADCs or trastuzumab/durvalumab were added to the cells in complete growth medium and incubated at 37 °C with 5% CO_2 for 96 h. Cell viability was determined using a CellTiter-Glo viability assay (Promega) according to the manufacturer's instructions. Cell viability was plotted as a percentage of that of untreated cells. Each measurement was taken in triplicate. Three independent replicates were performed. Data was processed using GraphPad Prism Version 9.3.0 and best-fit IC_{50} values of each compound were calculated using the log (inhibitor) vs response (variable slope) function. IC_{50}s are labelled "N/A" when it was higher than the range of concentrations examined.

Table of IC_{50}s

Table SI-6: The calculated IC_{50} values for trastuzumab **ADCs 1-3** in BT474, SKBR3 and MCF7 cell lines.

**Compound**	**IC_{50} (nM)**		
	**BT474**	**SKBR3**	**MCF7**
**ADC 1**	0.545	0.287	0.72
**ADC 2**	0.113	0.061	N/A
**ADC 3**	0.746	0.157	N/A

Table SI-7: The calculated IC_{50} values for durvalumab **ADCs 4-6** in MDA-MB-231 and MCF7 cells.

**Compound**	**IC_{50} (nM) MDA-MB-231**	**IC_{50} (nM) MCF7**
**ADC 4**	1.52	1.35
**ADC 5**	N/A	N/A
**ADC 6**	N/A	N/A

Cell Viability with Catalase Scavenger

Cells were seeded in 96-well plates for 24 h at 37 °C with 5% CO_2. MCF7 cells were seeded at 7,500 cells/well and MDA-MB-231 cells were seeded at 5000 cells/well. Cell media was then removed and replaced with 100 µL complete growth media containing catalase (400-1000 U/well) and incubated at 37 °C for 2 h. Then serial dilutions of ADCs in 100 µL complete growth media were added and cell growth was monitored on an IncuCyte® S3 Live-Cell Analysis System for 96 h with incubation at 37 °C and 5% CO_2. Cell viability was determined using a CellTiter-Glo viability assay (Promega) according to the manufacturer's instructions. Cell viability was plotted as a percentage of that of untreated cells. Each measurement was taken in triplicate. Two independent replicates were performed. Data was processed using GraphPad Prism Version 9.3.0 and best-fit IC_{50} values of each compound were calculated using the log (inhibitor) vs response (variable slope) function. Confluency increase was calculated using the equation: $[\frac{confluency_{final} - confluency_{initial}}{confluency_{initial}}] * 100$ whereby $confluency_{initial}$ is the % cell confluency at t = 3h after ADC treatment, and $confluency_{final}$ is the % cell confluency at approx. t = 96 h.

Table SI-8: The calculated IC_{50} values for durvalumab **ADC 1, ADC 4** in MDA-MB-231 and MCF7 cells with or without catalase.

Compound	IC_{50} (nM)			
	MCF7	MCF7 + CAT	MDA-MB-231	MDA-MB-231 + CAT
**ADC 1**	1.15	>10 nM	-	-
**ADC 4**	1.35	>10 nM	1.52	>10 nM

Quantification of extracellular hydrogen peroxide with Amplex™ Red

Amplex™ Red Hydrogen Peroxide/Peroxidase Assay Kit (Invitrogen) was used following the manufacturers instructions. Briefly, 20,000 cells/well were seeded in 100 µL F-12 Ham's nutrient mixture (for the no cell control, wells contained only F-12 Ham's nutrient mixture). After seeding for 24 h at 37 °C with 5% CO_2, 100 µL of the Amplex™ Red reaction mixture was added to each well and the fluorescence intensity monitored on a CLARIOstar plate reader over 30 minutes. Data shown is the reading at 30 minutes.

For catalase scavenging, after seeding the media was removed and replaced with Ham's nutrient mixture containing catalase (approx. 400-1000 U/well) (100 µL) and incubated at 37 °C, 5% CO_2 for 2h. Then, 100 µL of the Amplex™ Red reaction mixture was added to each well and the fluorescence intensity monitored on a CLARIOstar plate reader over 30 minutes. Data shown is the reading at 30 minutes.

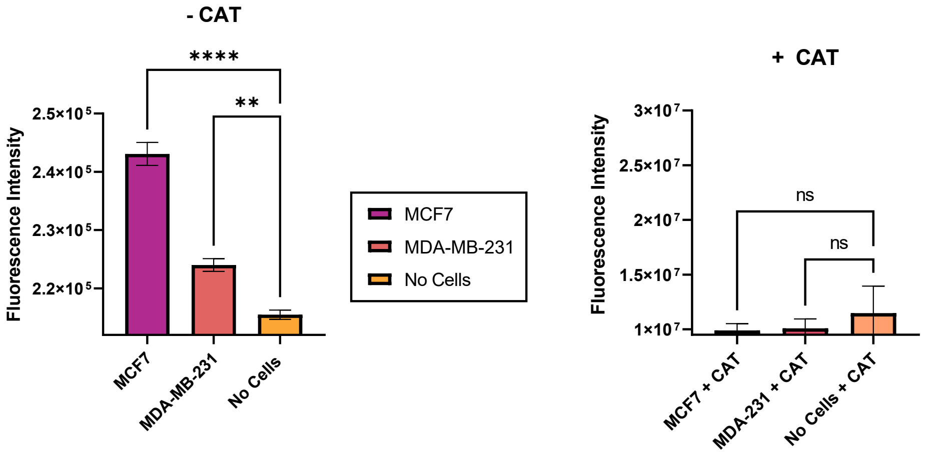

Figure S17: The fluorescence intensity from Amplex™ red reagent reaction with extracellular hydrogen peroxide of antigen-negative cell lines without scavenging of hydrogen peroxide (left) and with catalase scavenging (right). The fluorescence of Amplex red™ was compared to the fluorescence in the absence of cancer cells. Data was analysed by an ordinary one-way ANOVA whereby * = $p < 0.05$, ** = $p < 0.01$, *** = $p < 0.001$, **** = $p < 0.001$. ns = non-significant, $p > 0.05$.

NMR Spectra

4-(4,4,5,5-tetramethyl-1,3,2-dioxaborolan-2-yl)benzaldehyde (1a)

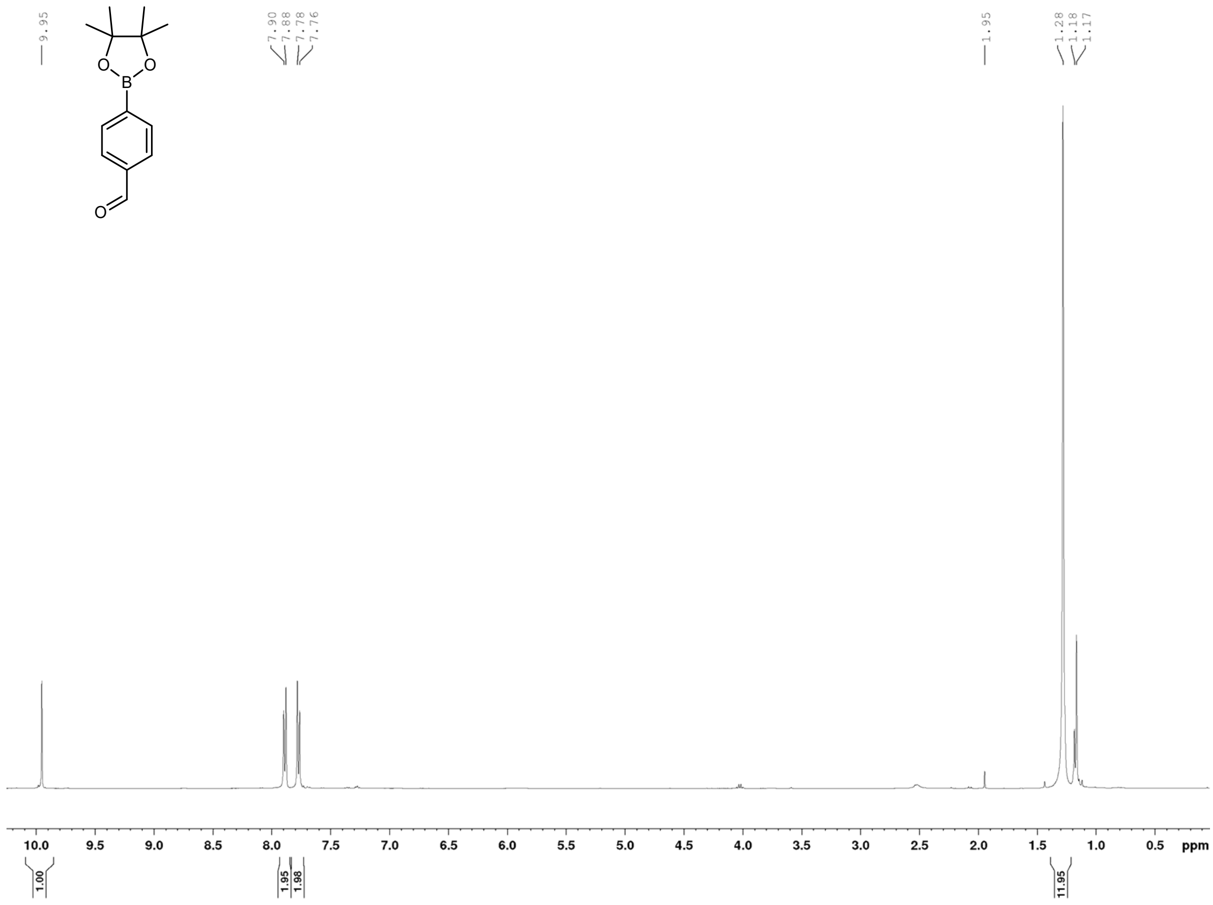

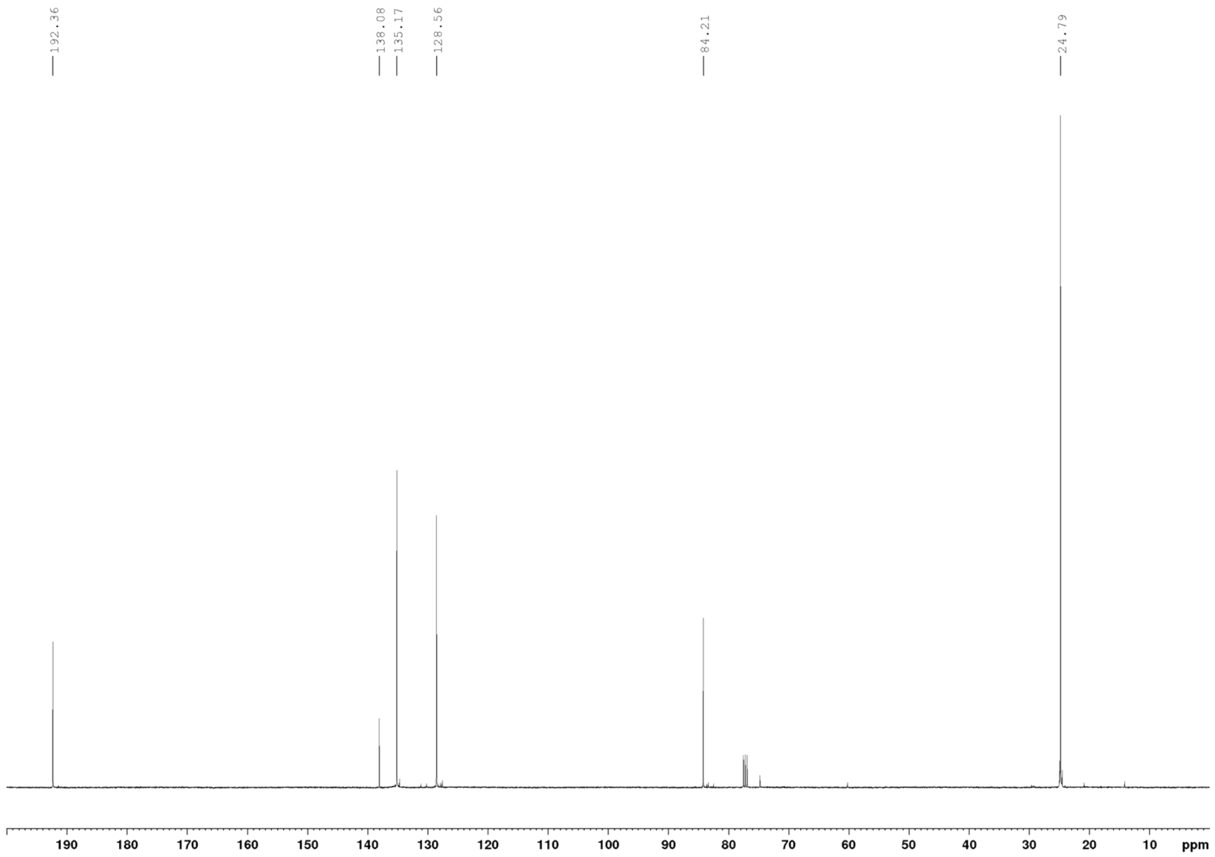

1-(4-(4,4,5,5-tetramethyl-1,3,2-dioxaborolan-2-yl)phenyl)but-3-yn-1-ol (1b)

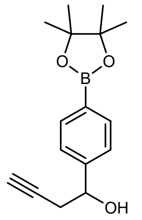

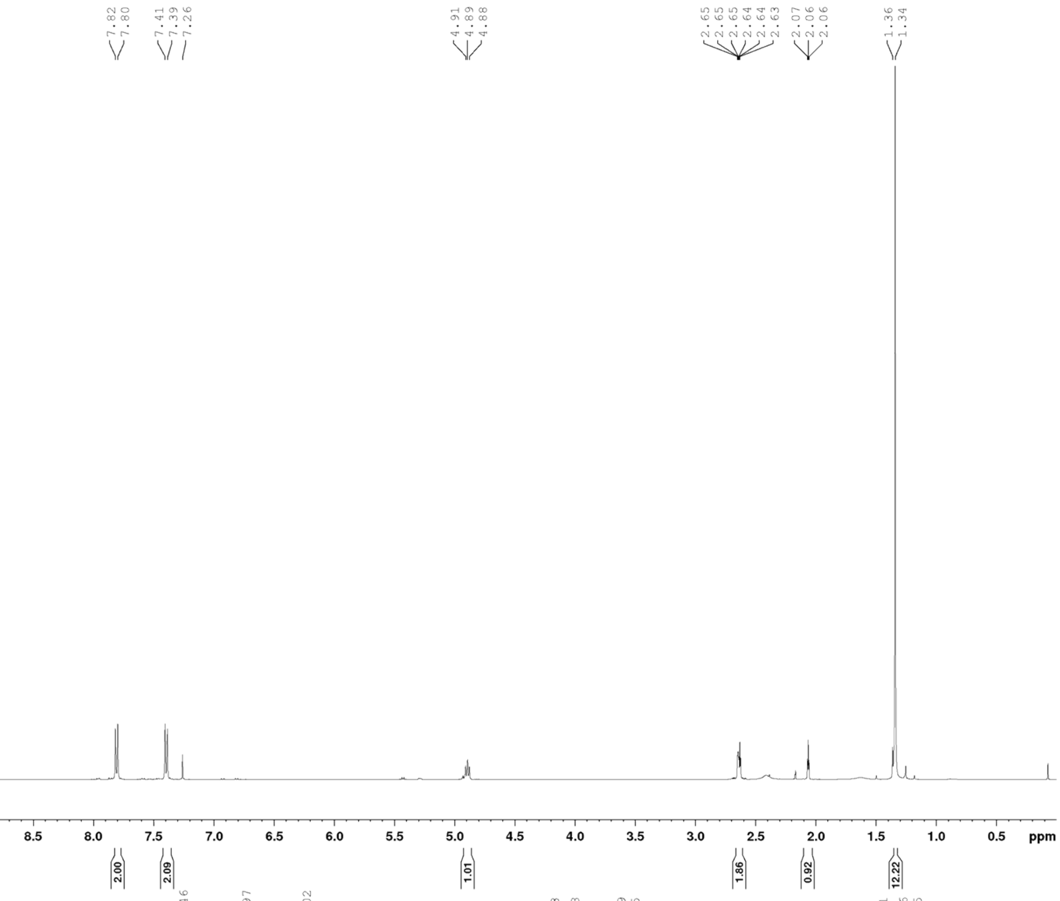

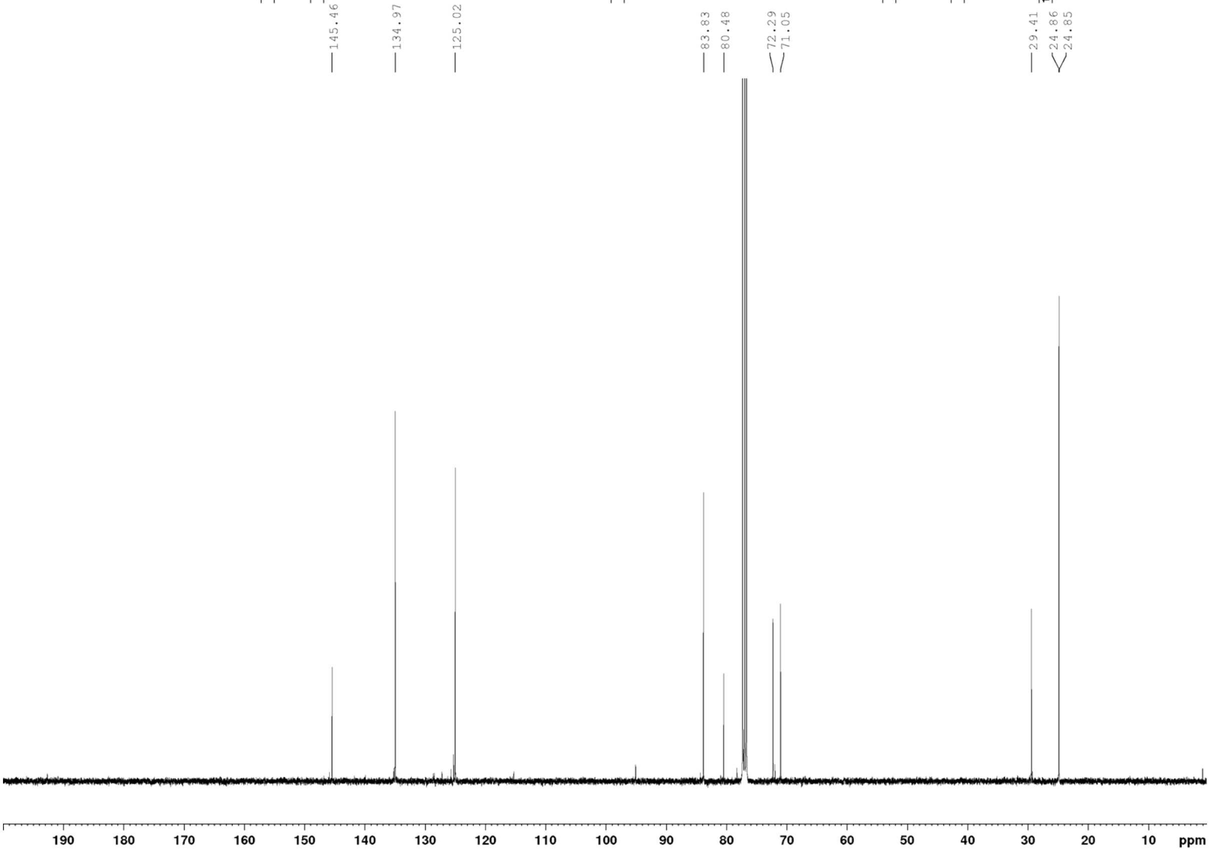

(4-(1-(((4-methyl-2-oxo-2H-chromen-7-yl)carbamoyl)oxy)but-3-yn-1-yl)phenyl)boronic acid (1c)

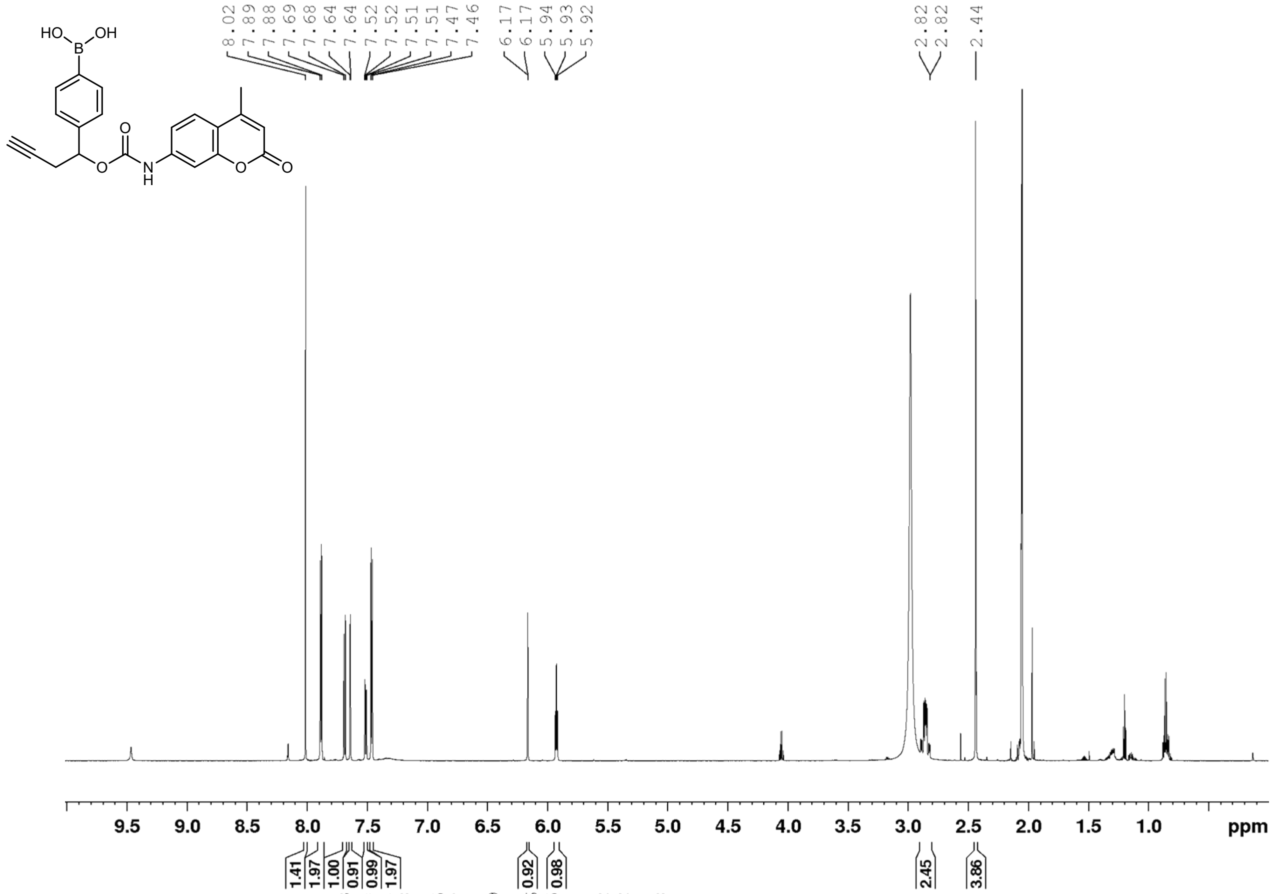
HO B OH
O
N H
O O
8.02 7.89 7.88 7.69 7.68 7.64 7.64 7.52 7.52 7.51 7.51 7.47 7.46
6.17 6.17 5.94 5.93 5.92
2.82 2.82 2.44
9.5 9.0 8.5 8.0 7.5 7.0 6.5 6.0 5.5 5.0 4.5 4.0 3.5 3.0 2.5 2.0 1.5 1.0 ppm
1.41 1.97 1.00 0.91 0.99 1.97 0.92 0.98 2.45 3.86

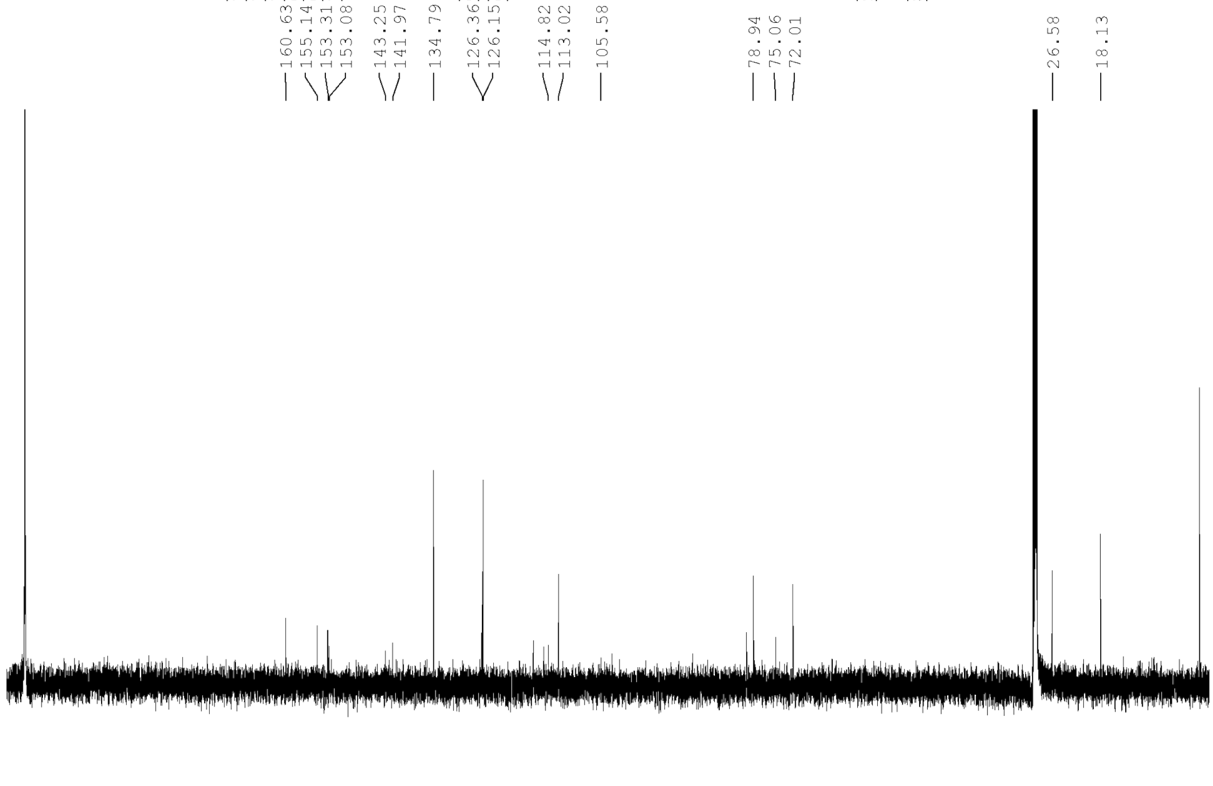
160.63 155.14 153.31 153.08 143.25 141.97 134.79 126.36 126.15 114.82 113.02 105.58
78.94 75.06 72.01 26.58 18.13
200 190 180 170 160 150 140 130 120 110 100 90 80 70 60 50 40 30 20 10 ppm

1-(4-bromophenyl)but-3-yn-1-ol (3b)

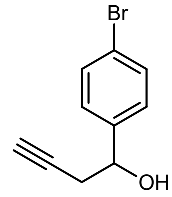

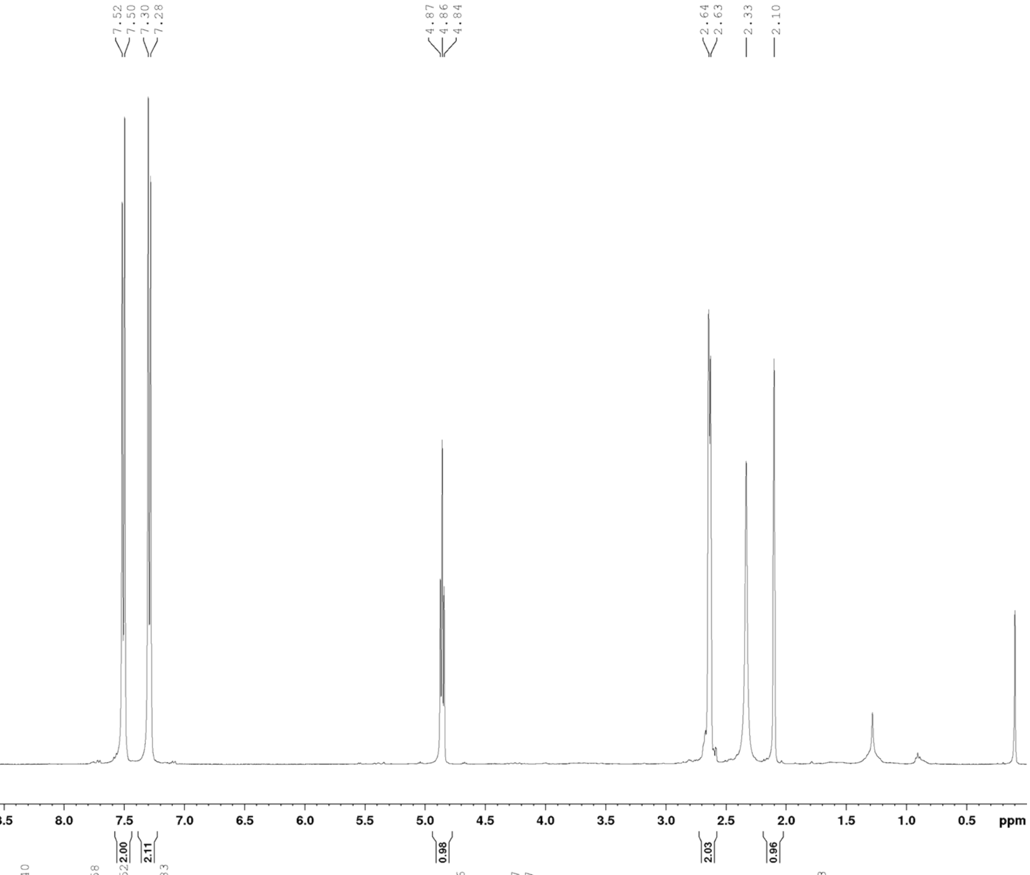

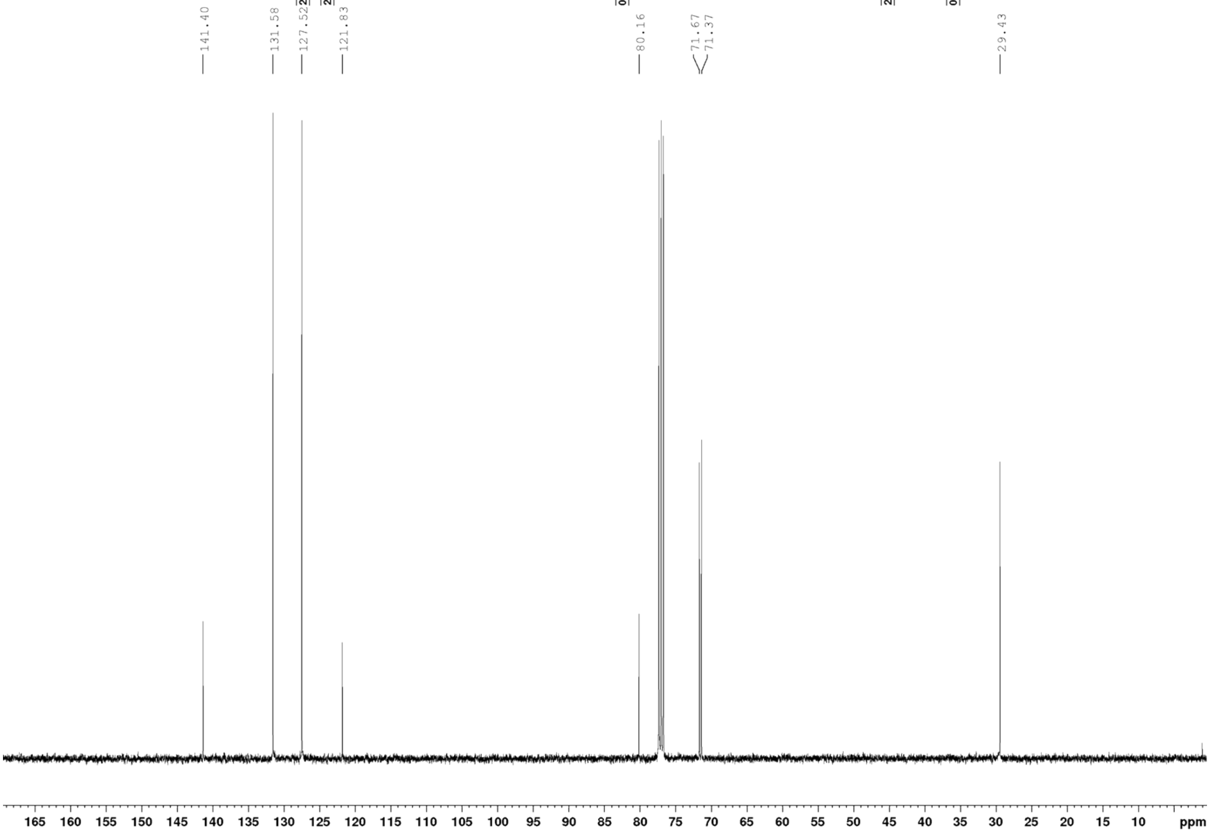

1-(4-bromophenyl)but-3-yn-1-yl (4-methyl-2-oxo-2H-chromen-7-yl)carbamate (3c)

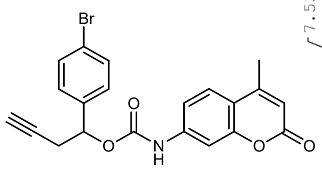

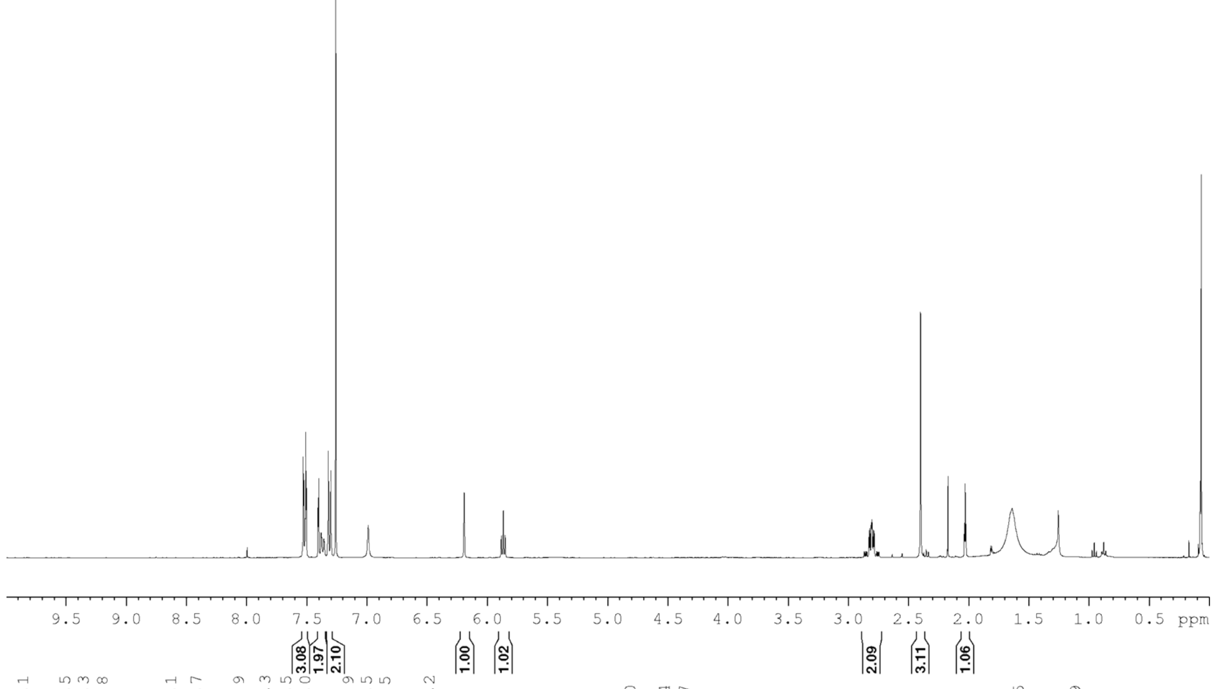

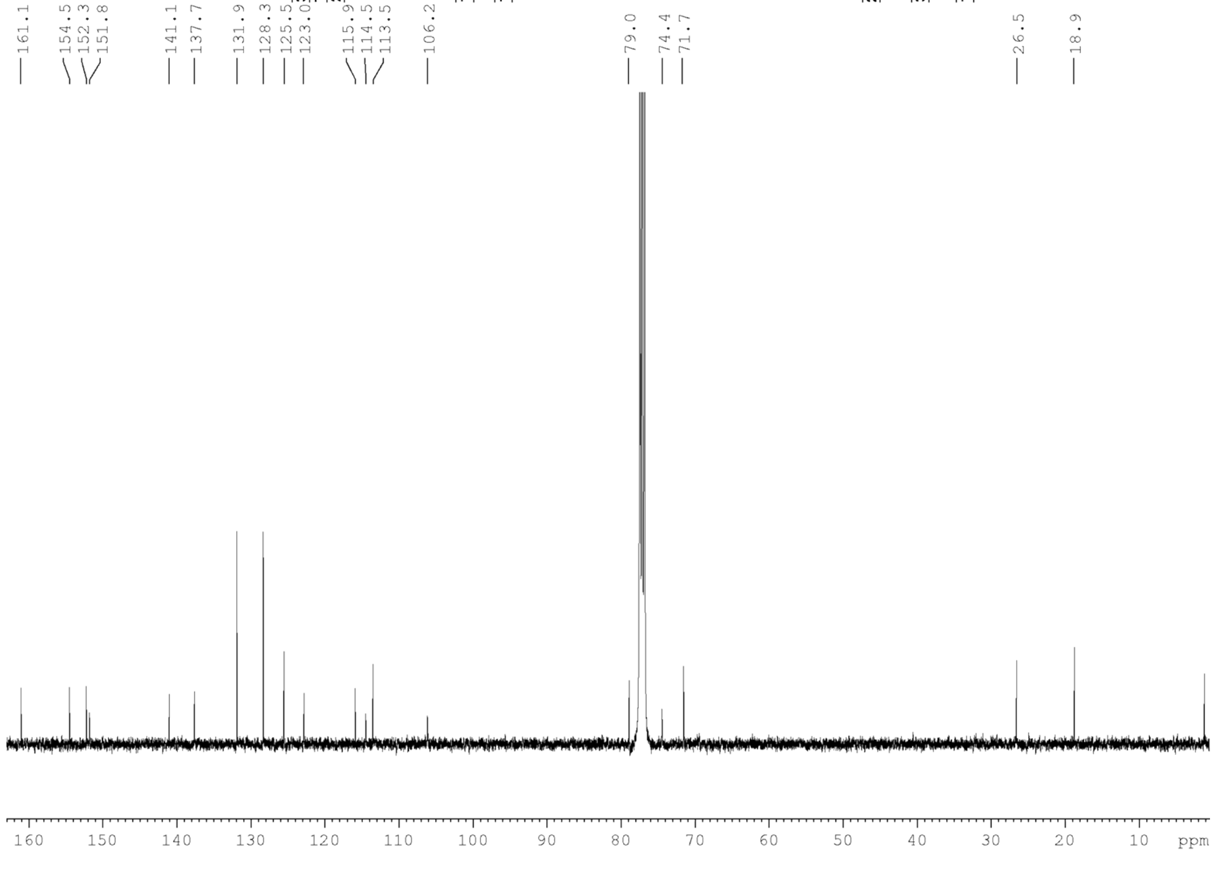

4-nitrophenyl (1-(4-(4,4,5,5-tetramethyl-1,3,2-dioxaborolan-2-yl)phenyl)but-3-yn-1-yl) carbonate (1d)

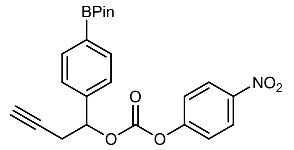

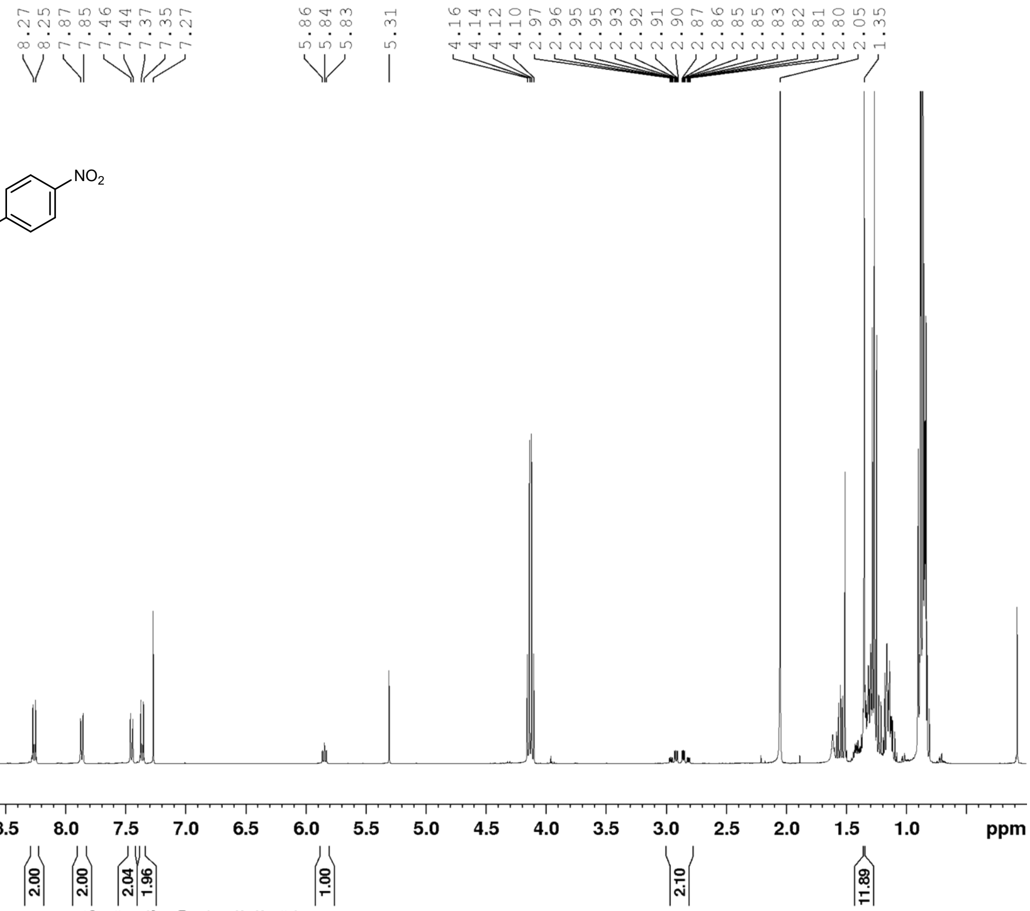

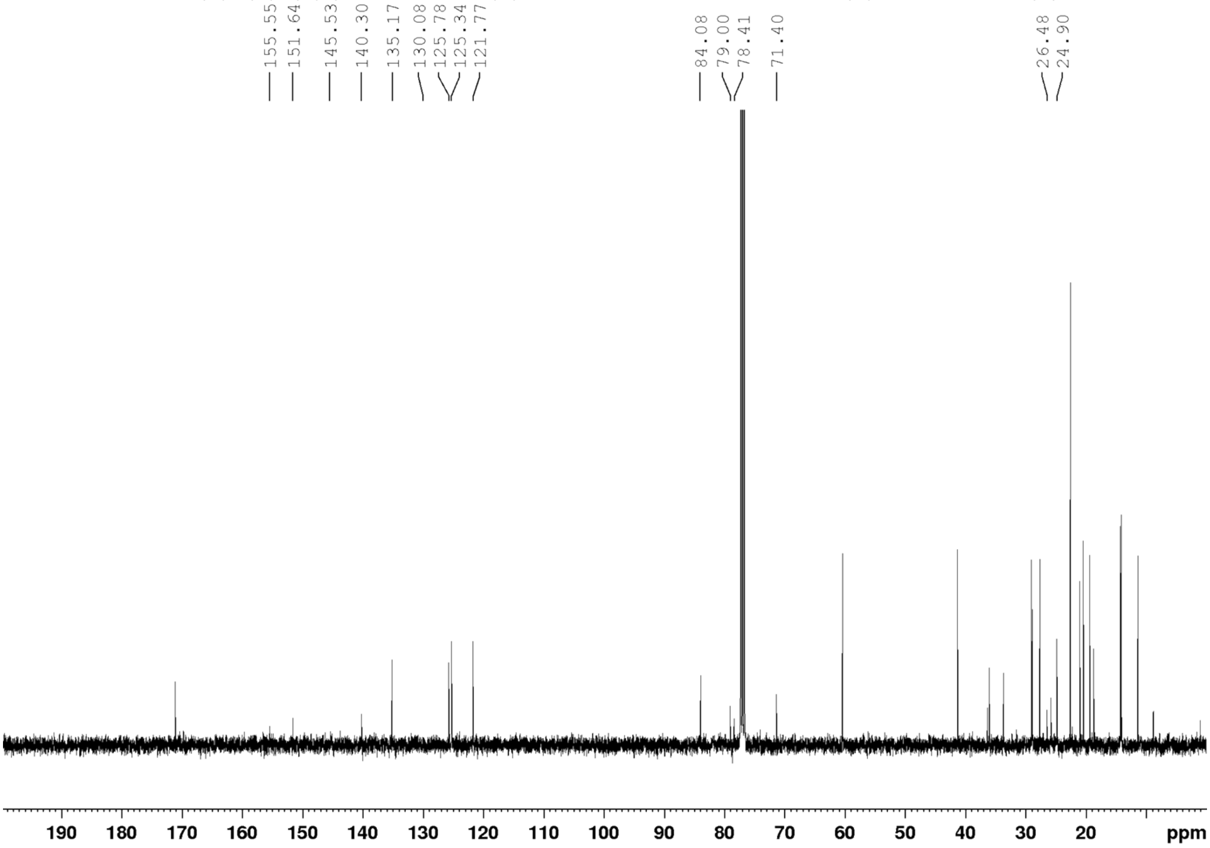

(S)-2-((((9H-fluoren-9-yl)methoxy)carbonyl)amino)-5-ureidopentanoic acid (2a)

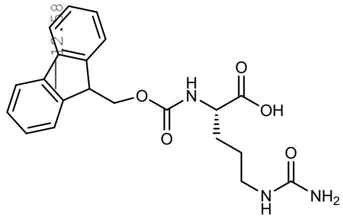

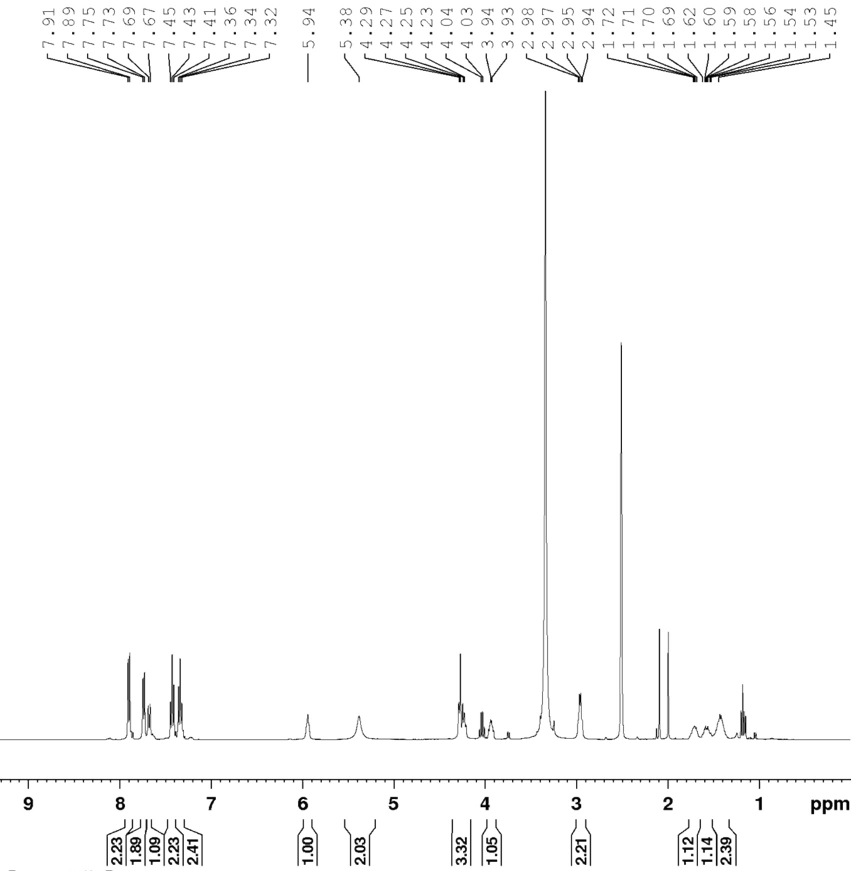

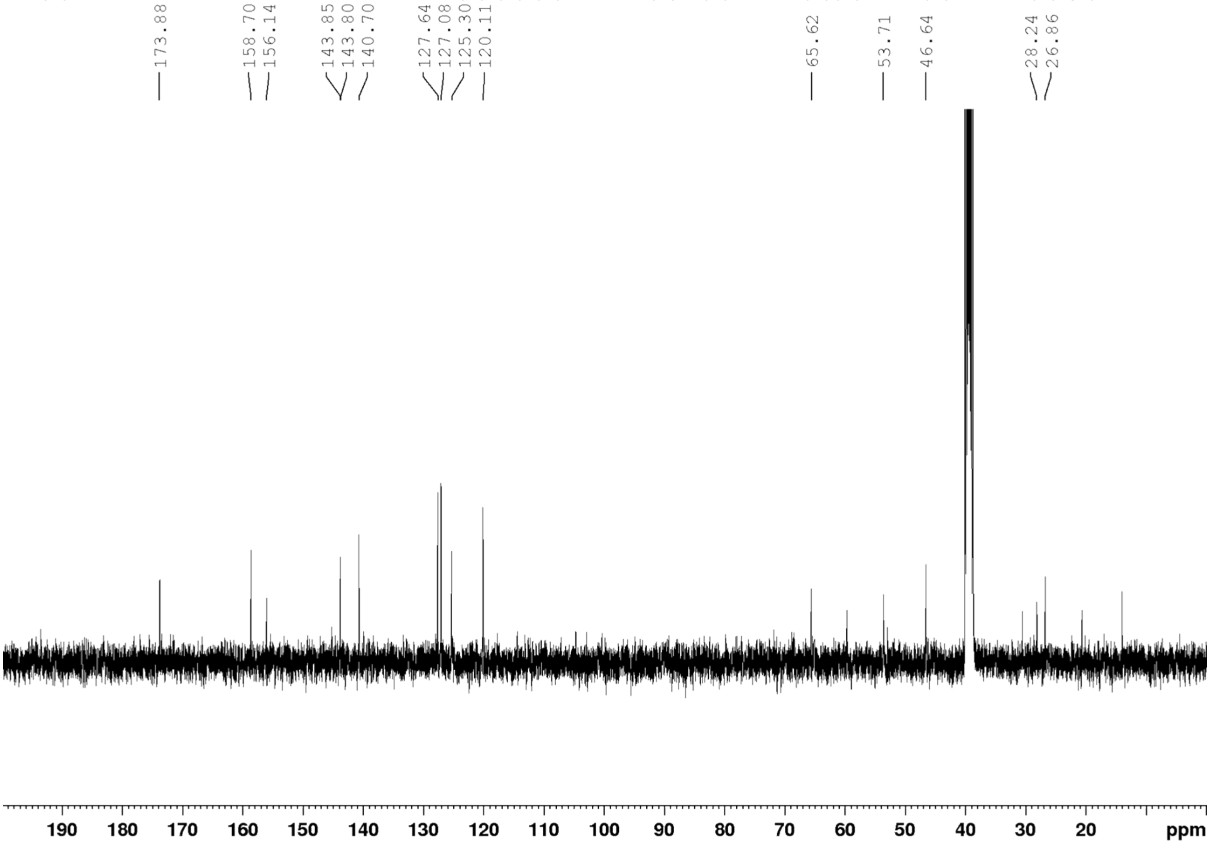

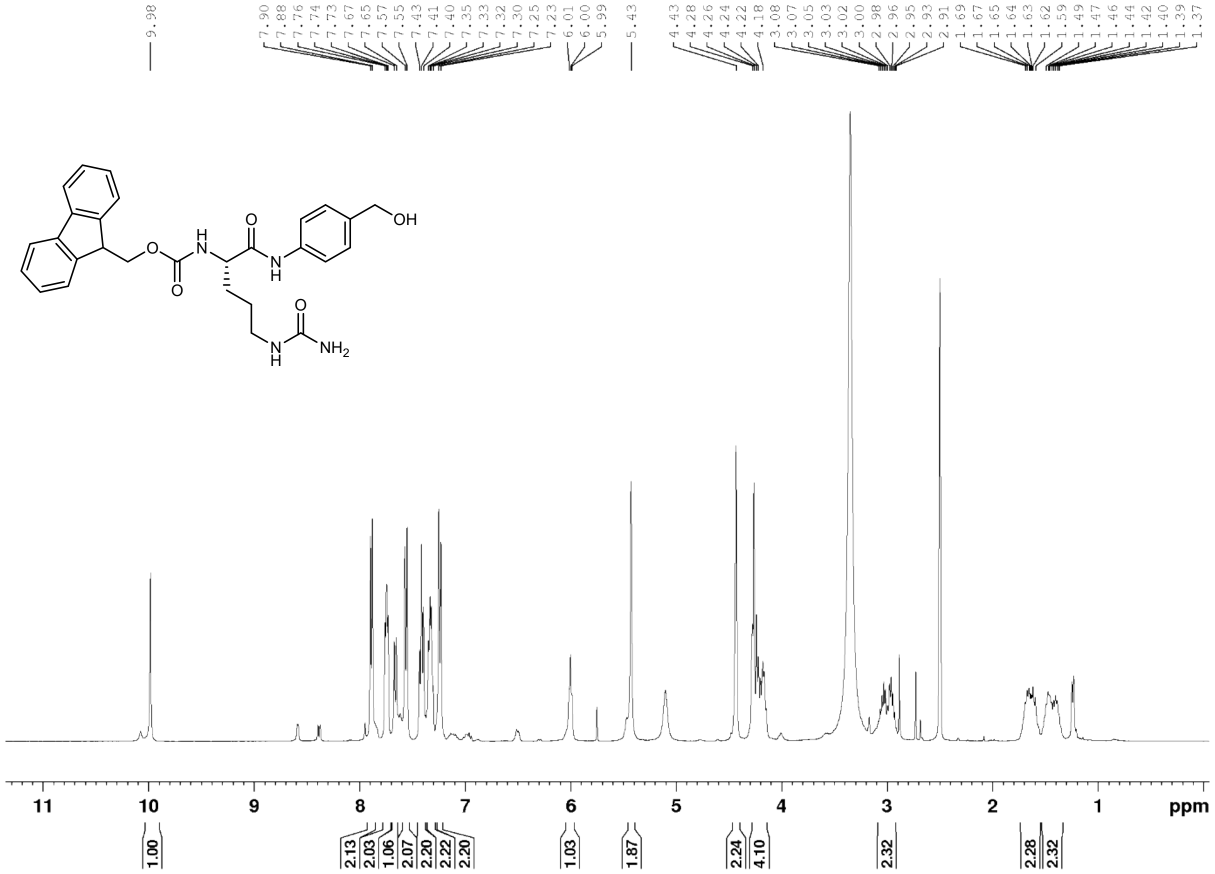

FMOC-Cit-PAB-OH (2b)

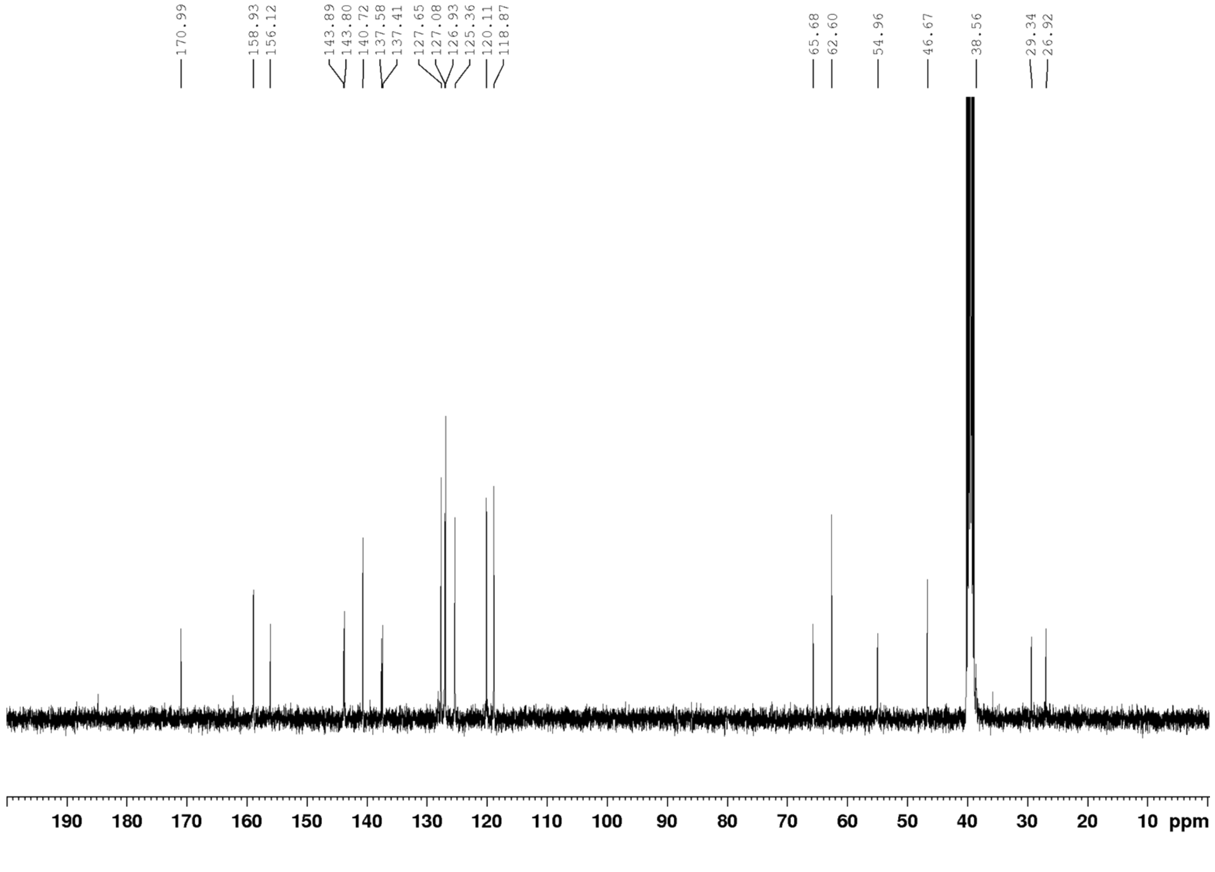

FMOC-Val-Cit-PAB-OH (2c)

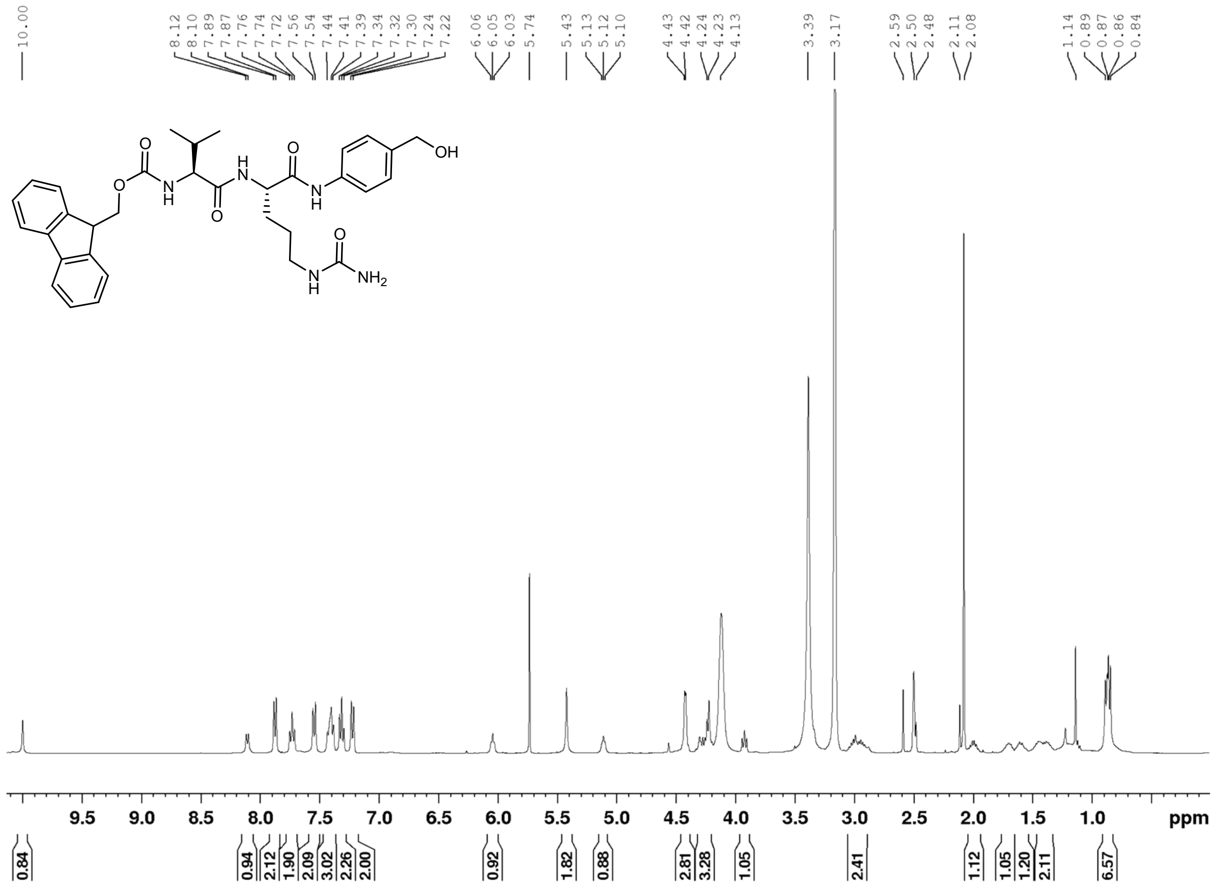

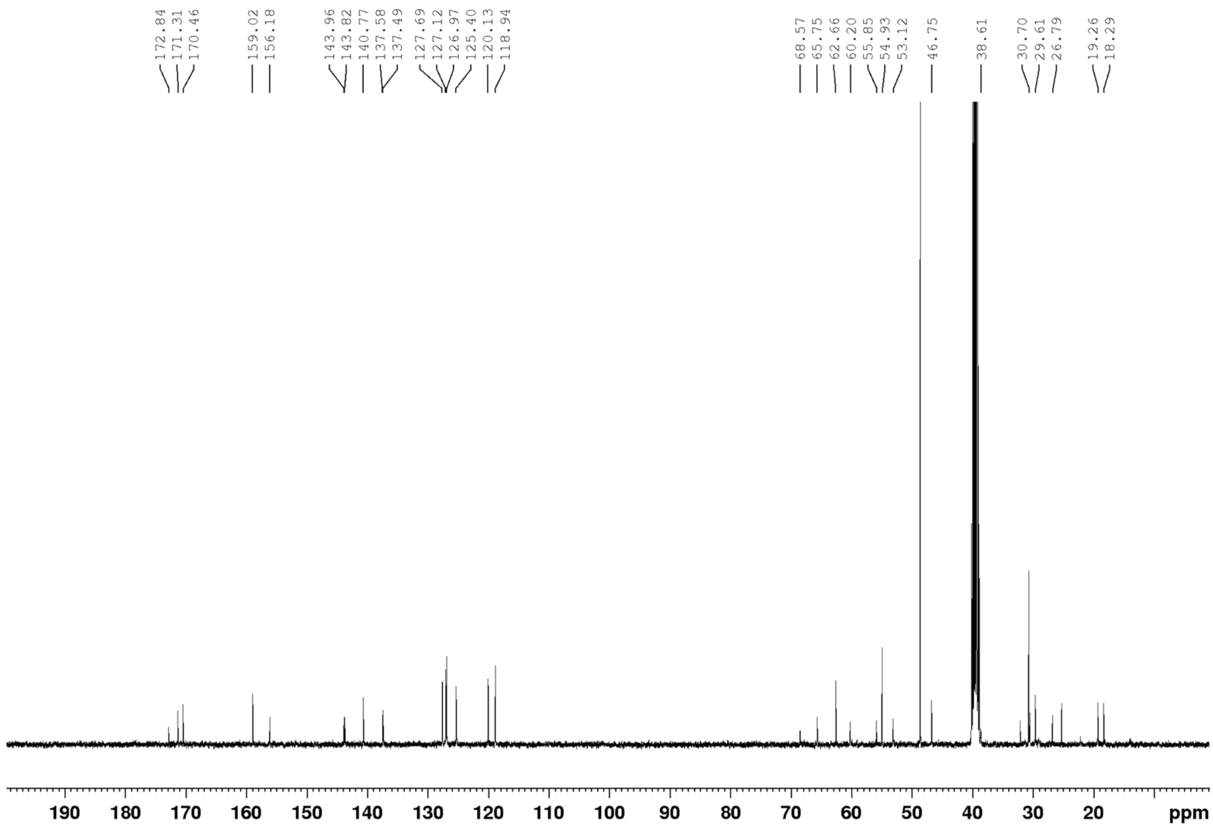

S)-*N*-(4-(hydroxymethyl)phenyl)-2-((S)-2-(2-methoxyacetamido)-3-methylbutanamido)-5-

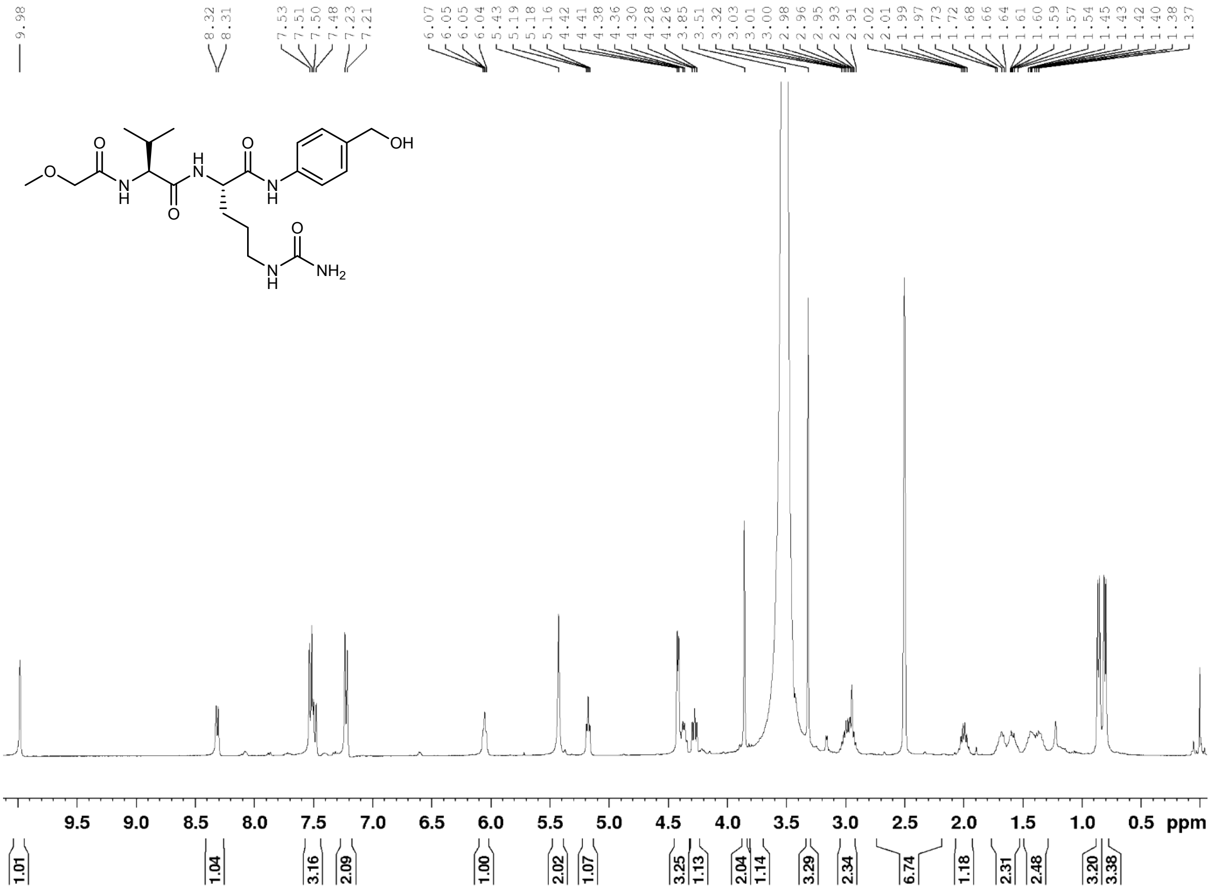

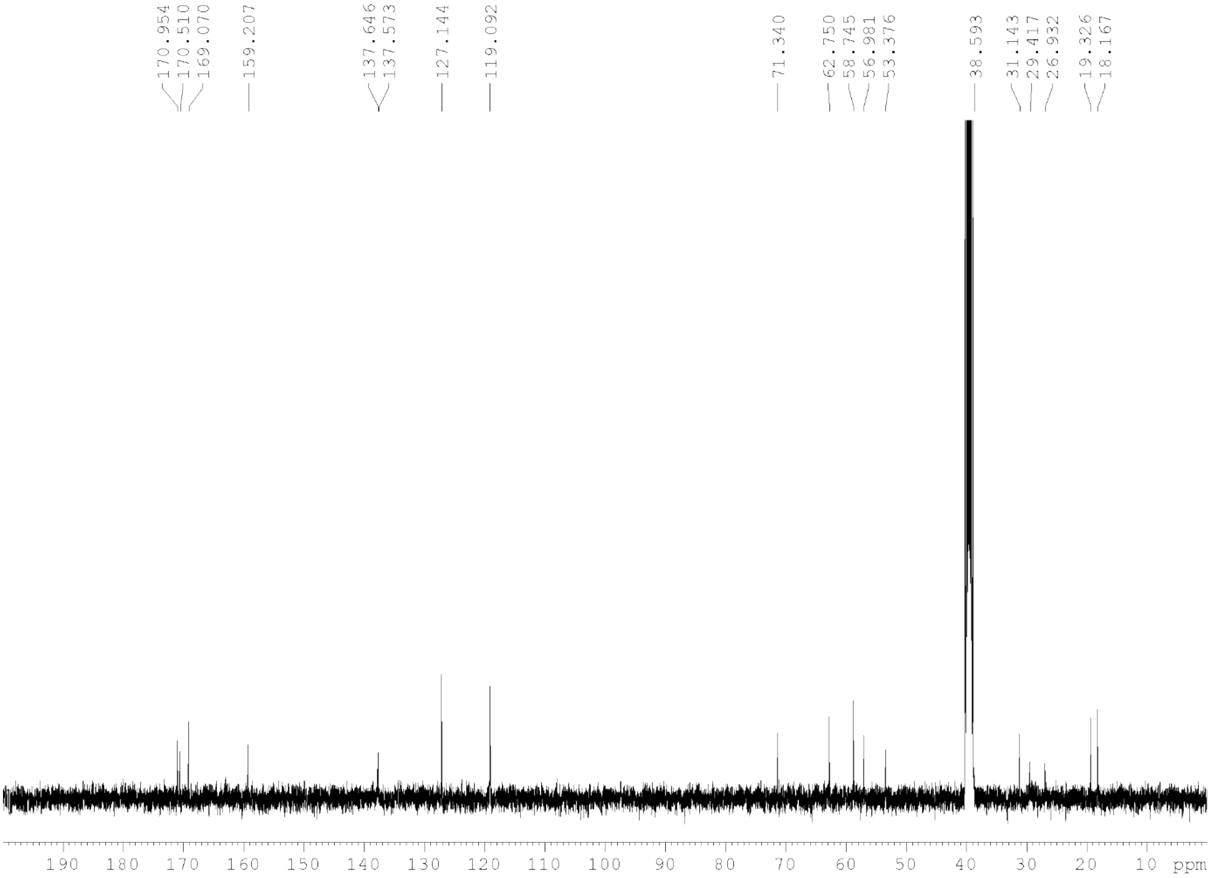

ureidopentanamide (2d)

4-((*S*)-2-((*S*)-2-(2-methoxyacetamido)-3-methylbutanamido)-5-ureidopentanamido)benzyl (4-methyl-2-oxo-2H-chromen-7-yl)carbamate (2e)

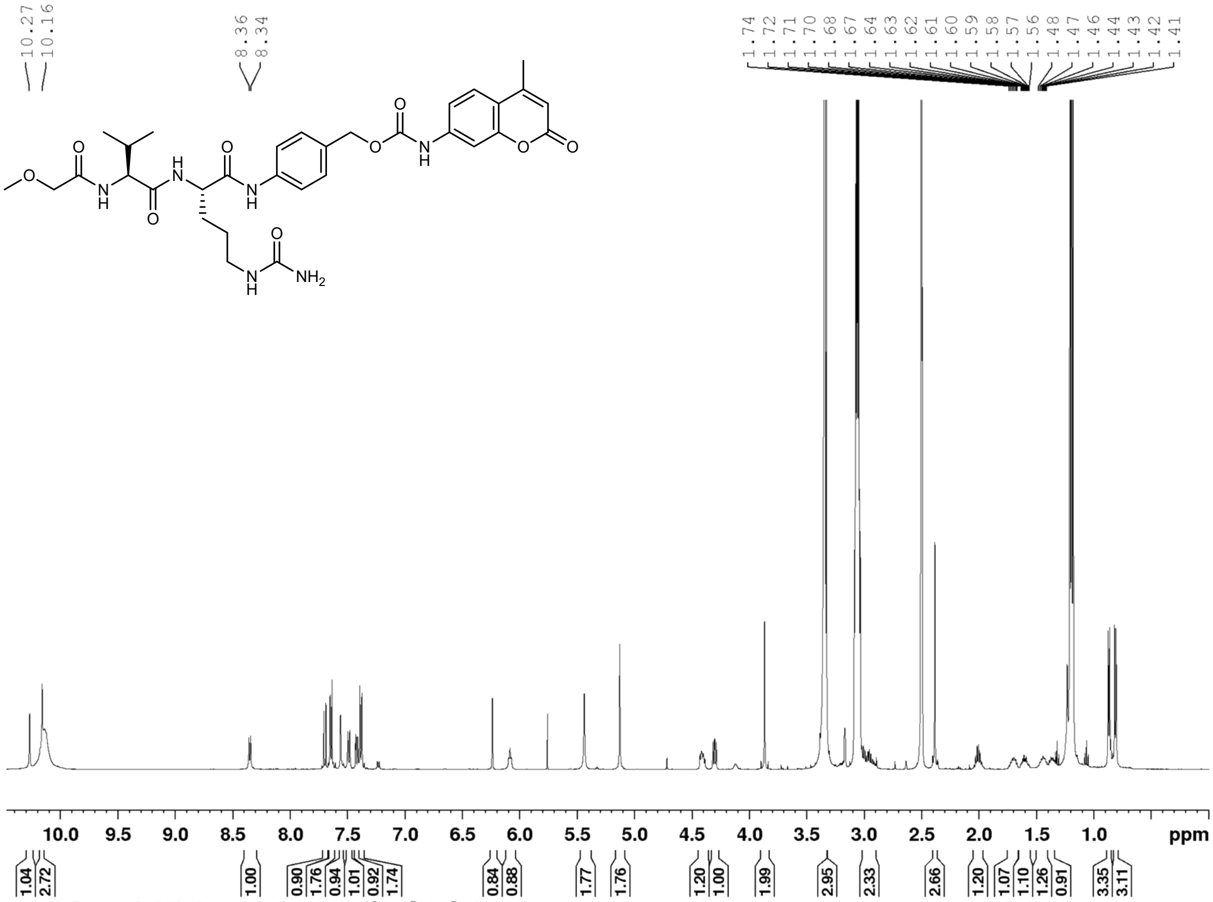
10.27
10.16
8.36
8.34
1.74 1.72 1.71 1.70 1.68 1.67 1.64 1.63 1.62 1.61 1.60 1.59 1.58 1.57 1.56 1.48 1.47 1.46 1.44 1.43 1.42 1.41
O
N
H
NH2
10.0 9.5 9.0 8.5 8.0 7.5 7.0 6.5 6.0 5.5 5.0 4.5 4.0 3.5 3.0 2.5 2.0 1.5 1.0 ppm
1.04 2.72 1.00 0.90 1.76 0.94 1.01 0.92 1.74 0.84 0.88 1.77 1.76 1.20 1.00 1.99 2.95 2.33 2.66 1.20 1.07 1.10 1.26 0.91 3.35 3.11

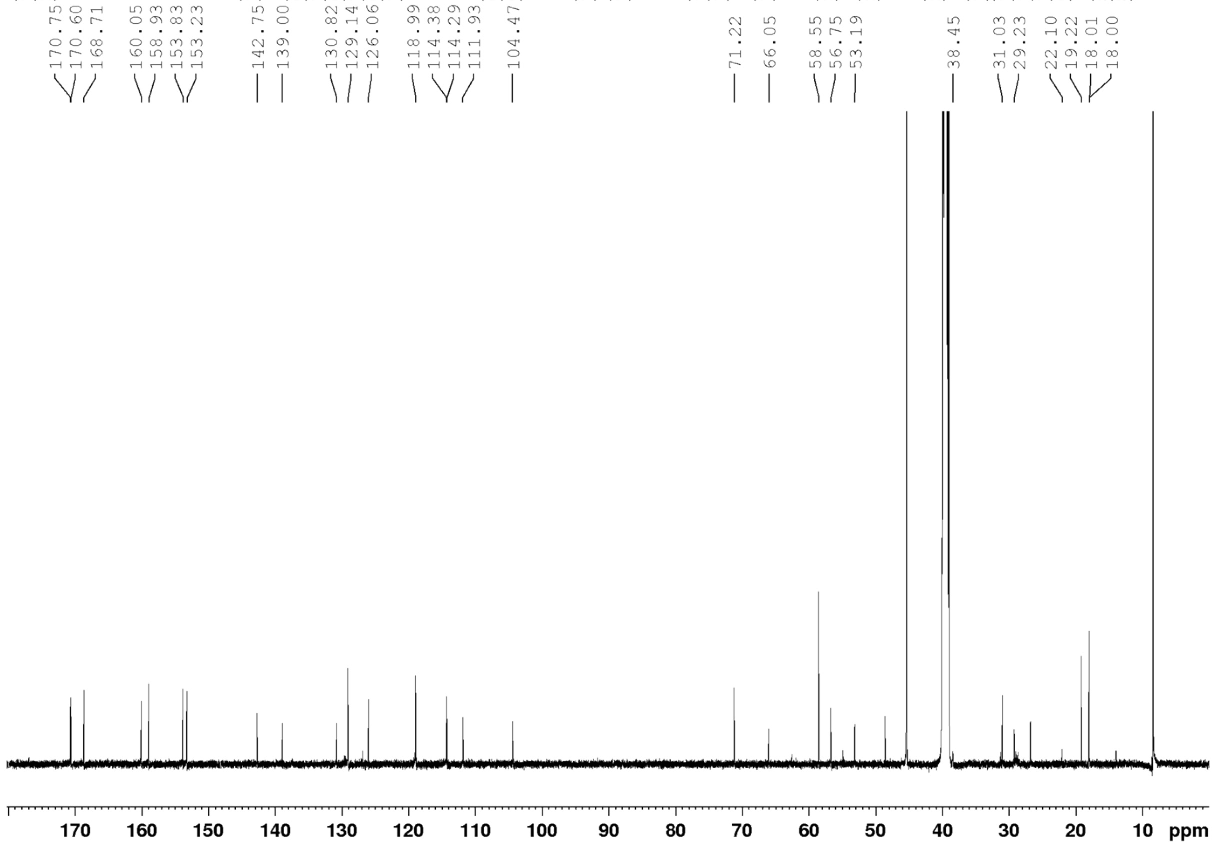
170.75 170.60 168.71 160.05 158.93 153.83 153.23 142.75 139.00 130.82 129.14 126.06 118.99 114.38 114.29 111.93 104.47 71.22 66.05 58.55 56.75 53.19 38.45 31.03 29.23 22.10 19.22 18.01 18.00
170 160 150 140 130 120 110 100 90 80 70 60 50 40 30 20 10 ppm
